# Reading-to-Writing Mediation model of higher-order literacy

**DOI:** 10.3389/fpsyg.2023.1033970

**Published:** 2023-06-30

**Authors:** Yusra Ahmed, Shawn C. Kent, Milena Keller-Margulis

**Affiliations:** ^1^Texas Institute for Measurement, Evaluation, and Statistics, University of Houston, Houston, TX, United States; ^2^College of Education and Behavioral Sciences, Houston Christian University, Houston, TX, United States; ^3^Psychological, Health, and Learning Sciences Department, University of Houston, Houston, TX, United States

**Keywords:** written expression, text writing, compositional fluency, reading comprehension, text reading fluency, learning disabilities

## Abstract

**Introduction:**

Writing difficulties frequently manifest comorbidly with reading challenges, and reading is implicated in particular acts of writing, such as reviewing and editing. Despite what is known, however, there remain significant barriers to understanding the nature of reading-writing relations, as few studies are comprehensive in the number and types of literacy skills evaluated. This study consists of a secondary data analysis of two studies employing structural equation modeling (SEM) to evaluate relations among reading and writing components skills independently, using the Direct and Inferential Mediation Model (DIME) of reading comprehension and Not-so-Simple View of Writing (NSVW) as theoretical frameworks.

**Methods:**

We examine relations between reading and writing components from these models with a sample of upper elementary students with/at-risk for learning disabilities (*n* = 405). Lower-order components included word reading, vocabulary, handwriting and spelling. Higher-order components included background knowledge, reading strategies, inferencing, planning, editing, and revision. The literacy outcomes were oral and silent reading fluency, reading comprehension, and writing quality and productivity. We systematically build a Reading-to-Writing Mediation (RWM) model by first merging the DIME and NSVW components in a direct effects model (Aim 1), expanding the joint model to include reading and writing fluency (Aim 2), evaluating indirect effects between DIME and NSVW component skills (Aim 3), and finally, evaluating indirect effects with reading and writing fluency (Aim 4).

**Results:**

The findings suggest that higher order fluency and comprehension skills are differentially related to writing activities and products.

**Discussion:**

The pattern of results helps elucidate the mechanisms of how various reading and writing skills transfer and relate. The results have implications for targeted and implicit instruction in multicomponent interventions and the use of screeners to identify areas of risk.

## Introduction

National data on student performance in the United States indicates that reading and writing (R-W) continue to be areas of concern, particularly for children with or at-risk for learning difficulties. Just over one-third of the nation’s students in grades 4, 8, and 12 demonstrated proficient reading comprehension in the latest National Assessment of Educational Progress (NAEP; McFarland et al., 2019). The NAEP Oral Reading Study (White et al., 2021) showed that reading fluency is also a concern for grade 4 students with reading difficulties; specifically, students who performed at the basic or below basic level on the NAEP reading assessment performed significantly lower on measures of passage fluency, accuracy, and expression in comparison to students at the proficient and advanced reading levels. Students at the lowest level of performance averaged just 71 words read correct per minute and 82% accuracy. Historically, an even lower percentage (~25%) of students have attained proficiency in writing (Aud et al., 2012). Beyond the K-12 setting, writing serves as a gatekeeper to college access for underrepresented students. Of note, 58% of employers rated recent graduates as not proficient in writing, and proficiency in written communication skills was considered essential by nearly 96% of employers, who report often considering writing skills when making decisions about hiring and promotions (National Association of Colleges and Employers, 2017). The National Commission on Writing (2004, 2005) estimated that $3.1 billion are spent annually remediating writing skills in the private sector and $250 million in the public sector.

Many individuals who have difficulty with reading also have challenges in the area of writing, highlighting the established connection across these skill areas; however, there is limited research on the relationships among R-W component skills. One group of students particularly likely to demonstrate lower performance in R-W are those with learning difficulties (LD; Fletcher et al., 2018). The most commonly occurring difficulties for students with LDs are word reading, fluency, and comprehension. Students with word-level reading difficulties, such as those with dyslexia, exhibit difficulties not only with handwriting and spelling but also demonstrate deficits with composition skills such as editing (e.g., Berninger et al., 2008; Carretti et al., 2016; Hebert et al., 2018). Difficulty with reading comprehension has been linked to difficulty with composition quality (Cragg and Nation, 2006; Re and Carretti, 2016). Given this reality, and the need to better understand the R-W connection in this group, the primary purpose of this study was to conduct an exhaustive examination of the associations among R-W skills to better inform research and practice. Theoretical and empirical accounts of literacy (described below) suggest that multiple skills contribute to the inter-connectedness of R-W. In an era when multiple interventions are available and easily accessible, it is important for researchers and practitioners alike to understand the complex patterns in which literacy skills interact with each other and how reading skills can be leveraged to explicitly teach skills in the academic domain of writing (and vice versa). Understanding the connections across these skill areas is critical given not only the opportunities for better conceptual or theoretical understanding of the relationships but also the potential for direction regarding the provision of literacy supports broadly.

## Reading-to-Writing directionality

The instructional context in which R-W skills are taught can significantly impact the connection between reading and writing, as writing is both shaped and constrained by socio-cultural factors (Graham, 2018). Research has demonstrated that when R-W skills are integrated and taught together, rather than in isolation, students develop stronger R-W skills (Hebert et al., 2018). For example, writing-to-learn approaches emphasize using writing as a tool for understanding and learning new information through activities like summarizing or creating concept maps, which can help students organize and make sense of texts, which in turn can improve reading comprehension. R-W instruction can also be integrated through reading-to-write approaches, which emphasize using reading as a tool for developing writing skills. When students read a wide range of texts, they are exposed to a variety of text genres and structures, which can improve their own writing skills. We focus on the Reading-to-Writing directionality because the instructional context of this study was business-as-usual (i.e., reading instruction was more easily and frequently implemented than writing instruction; see Ahmed et al., 2022a). While we acknowledge that directionality of influence between R-W is not necessarily unidirectional, we emphasize the need for careful consideration of contextual factors, such as the nature of R-W instruction, orthography, and other relevant factors, in determining the directionality of the influence of one set of skills on another. In this study, we present an alternative Writing-to-Reading model in Supplementary Appendix B to acknowledge the potential for bidirectional influence, noting that the current study is limited in its capacity to establish causal connections because doing so necessitates an experimental research design. In the next sections, we start by presenting piecemeal evidence of R-W associations from experimental and correlational studies and end with component skills models that incorporate multiple skills and their interrelations.

## Word-, sentence-, and text-level reading-writing

Robust relations between word reading and transcription skills (handwriting and spelling) have been demonstrated (e.g., Berninger et al., 2002; Abbott et al., 2010; Georgiou et al., 2020), and word-level literacy has been established as an important precursor of production and quality of writing. In addition, there is ample evidence for the relation between reading comprehension and writing skills, including word-level skills like spelling (Berninger et al., 2002) and text-level writing outcomes (e.g., Cragg and Nation, 2006; Carretti et al., 2016). There is little evidence, however, of the relationship between oral and silent reading fluency and the various levels of writing performance at the letter/word, sentence, and discourse levels.

Automaticity in R-W is a general issue affecting children with LDs, although little is known about whether rate-subtypes of disability can be reliably identified as separate subgroups of LD (Fletcher et al., 2018). Compromised accuracy and automaticity of word-level skills result in problems of automaticity at the sentence and discourse level of R-W fluency by reducing access to processes required for constructing meaning (e.g., inferencing or revision), as conscious attention to decoding or spelling makes R-W slow and laborious (Fletcher et al., 2018). Consequently, children with LDs are limited to proofreading texts for mechanics but not substance or content (MacArthur, 2016).

Oral reading fluency (ORF)—the ability to read aloud with speed, accuracy, and proper expression— is heavily used both in research and practice as an overall indicator of performance in reading because it is highly predictive of reading problems in children with LD (Deno, 2003). ORF is an overall indicator of reading performance in early elementary grades where the number of words read correctly in 1 min is the outcome observed. Measures of ORF are used as part of screening efforts in the context of multi-tiered systems of support (MTSS) where performance in reading is measured periodically and used to identify those students at-risk for poor performance as well as to monitor progress in response to instruction or intervention.

Limited studies investigate the relationship between reading fluency and writing —specifically, planning, translating, and revising—in typically developing and children with LDs (Graham and Hebert, 2011) despite the clear correlations of reading fluency with writing outcomes and the regular use of ORF in practice (e.g., Shinn et al., 1992; Fewster and MacMillan, 2002; Cragg and Nation, 2006; Berninger et al., 2008; Codding et al., 2015). There is support for the notion that the rate, accuracy, and prosody in ORF may relate to spelling at the word-level (Bear, 1991; Lefly and Pennington, 1991; Ritchey and Coker, 2014), although other researchers have found that ORF did not relate to spelling after controlling for other foundational reading skills (Morris et al., 2017). Two studies found that ORF was related to the total number of words written at the text-level for children in elementary grades (Ahmed et al., 2014; Tortorelli and Truckenmiller, 2023), but these studies did not explore the relations of ORF with sentence-level writing or text-level writing quality. Bear (1991) hypothesized that ORF may play a role in one writing process (planning) because word- and phrase/sentence-level planning are especially evident in oral expression (e.g., phrasal intonation and placement of accent in reading unfamiliar words). However, von Koss Torkildsen et al. (2016) found that ORF was *not* related to another writing process (revision) after controlling for executive function (working memory and attention) and spelling. To better understand individual differences in R-W, a more complete understanding of the role of ORF is necessary, when individuals engage in foundational writing skills at the word-level (e.g., spelling), self-monitoring during writing processes at the sentence-level (e.g., editing), and general writing outcomes at the text-level (quality and productivity).

Silent reading fluency (SRF) —the ability to read silently with speed and comprehension—emerges as a more important skill as students progress to higher grade levels, and ultimately adulthood, because SRF is required for more advanced texts. It is possible that SRF plays a more critical role in written expression when children are in the transitional phase from ORF to SRF and when the focus of instruction shifts from sentence- to text-level (Bear, 1991; Berninger et al., 2013; van den Boer et al., 2022). Notably, children with LD exhibit deficits in SRF that are commensurate with, or more pronounced than, their deficits in ORF (van den Boer et al., 2022). Research shows that SRF training results in better spelling for children with dyslexia (Berninger et al., 2013). Further, SRF contributes to children’s ability to revise sentences, and revision also impacts SRF (Ahmed et al., 2014). To our knowledge no studies have systematically examined the contributions of both ORF and SRF to higher-order writing processes (Shanahan, 2012). We propose that when evaluating sentence level R-W together with word- and text-level R-W, sentence level skills will have greater predictive power for text level writing quality and fluency than word level literacy.

## Higher-order reading-writing connections

Reading skills are needed when individuals engage in self-monitoring during the planning, revision, and reviewing states of writing. That is, one’s ability to accurately and efficiently decode, scan, and comprehend what has been written are pre-requisite skills for revising the composition (McCutchen, 1996). Fitzgerald and Shanahan (2000) outline four areas of shared knowledge: (a) content or domain knowledge; (b) meta-knowledge about written language (i.e., functions and purposes); (c) pragmatic knowledge of text attributes (e.g., words, syntax, and usage); and (d) procedural knowledge for accessing information purposively, setting goals, analyzing, etc. For example, text is extended according to background knowledge and the writer’s hypotheses about the readers’ knowledge (Flower and Hayes, 1980), particularly in later grades, when students are required to write about topics outside of themselves (Davis and Winek, 1989). Knowledge of text structures help students understand the purpose for presenting information, the organization of ideas, and the use of similar schema across texts. A meta-analysis of 45 studies (Hebert et al., 2016), showed that text structure instruction (measured as strategies, such as evaluation of text) improved expository reading comprehension, particularly when including writing in that instruction. Furthermore, it has long been recognized that vocabulary plays a key role in writing development (Olinghouse and Wilson, 2013) with significant relationships evident for vocabulary to spelling and to planning before writing (Vanderberg and Swanson, 2006). Vocabulary knowledge is also related to individuals’ written production and text quality (e.g., Carretti et al., 2016; Kim and Schatschneider, 2017). Finally, planning involves goal-setting and knowledge mobilization, requiring students to evaluate their own knowledge of the topic, and narrow their topics and goals (Tierney and Shanahan, 1991).

Older theoretical models devoted solely to the interaction among R-W processes (Pearson and Tierney, 1984; Langer, 1986) and broader frameworks of writing in adults also specify various mechanisms of co-development (Bereiter and Scardamalia, 1987; Tierney and Shanahan, 1991; Fitzgerald and Shanahan, 2000; Deane et al., 2008). For example, inferencing allows writers to elaborate a new representation from a former one and is related to writing for children in first grade (Kim and Schatschneider, 2017) and in college (Connelly et al., 2006). Overall, higher-order reading skills (background knowledge, inferencing, strategies for reading) predict writing-specific processes such as planning, editing, and revising (e.g., Tierney and Pearson, 1983; Kirby et al., 1986; Singer and Bashir, 2004; Weston-Sementelli et al., 2018). These reading skills are also related to the quality of written composition (e.g., Fitzgerald and Shanahan, 2000; Decker et al., 2016; Kim and Schatschneider, 2017; Weston-Sementelli et al., 2018). The important conclusion from the theoretical literature is that higher-order reasoning processes of R-W are text-based (i.e., require interaction with text).

## Component-skills models of reading and writing

To examine the above-mentioned R-W relationships, it is important to situate the study within the specific component models of R-W focused on in the present study. Although several models exist in both areas, there is significant overlap in the component skills represented in each. For the current study, we chose to frame our examination of the R-W relationship using the Direct and Inferential Mediation Model (DIME; Cromley and Azevedo, 2007) and the Not-so-Simple View of Writing (NSVW; Berninger and Winn, 2006) because they are well aligned with cognitive theories of reading and writing, respectively.

### Direct and Inferential Mediation model of reading comprehension

The DIME model posits that the several components work together for the end goal of comprehension, and account for virtually all the variance in reading comprehension (Cromley and Azevedo, 2007; Ahmed et al., 2016). The elements of the DIME model include: (1) *Decoding* and (2) *Vocabulary*, because students who have adequate word reading skills and word knowledge can better understand text (Hoover and Gough, 1990), (3) *Background knowledge*, because readers who possess high levels of general knowledge perform better on reading comprehension and retain the information for longer periods of time (Chiesi et al., 1979; Kintsch, 1988), (4) *Inferences* (knowledge-to-text and text-to-text integration) are automatically generated when students understand what is implied by the text without explicitly being stated (Cain and Oakhill, 1999; Barnes and Dennis, 2001), and (5) *Reading strategies*, refers to engagement in cognitive and meta-cognitive strategies, such as summarizing, structuring, drawing conclusions, and evaluating text (O’Reilly and McNamara, 2007). The DIME model can be seen as an extension of the Simple View of Reading (SVR; Gough and Tunmer, 1986). While the SVR conceptualized comprehension as a product of word reading/decoding and oral language/linguistic comprehension, in the DIME model, the components of linguistic comprehension are further specified as lower (i.e., word reading, vocabulary) and higher-level (background knowledge, strategies for reading, inferencing) component skills; reading comprehension is thought to be influenced directly and indirectly via these skills. To enhance understanding of relations among the DIME components, four studies included silent reading fluency (SRF) or efficiency as an additional predictor (Smith, 2013; Oslund et al., 2016, 2018; Völlinger et al., 2018). In general, SRF was a strong predictor of reading comprehension for children in upper elementary or middle school, but vocabulary had the largest direct effect, followed by inferences. The relation of SRF and comprehension was dependent on reader proficiency.

### Not-so-Simple View of Writing

An early component skill model of writing was the simple view of writing, which posited that transcription and ideation (i.e., text generation) together were necessary for writing (see Juel et al., 1986; Berninger et al., 2002). As a follow-up and extension, the NSVW holds that transcription skills (e.g., spelling and handwriting fluency), along with ideation, interact with higher-order, executive, and self-regulatory functions to produce writing through planning, composing, and revision. That is, proficient writers possess linguistic knowledge of grammar and syntax to create coherent and well-structured sentences and also engage in multiple cycles of reviewing, revising, and editing their work to improve their content, organization, and language use. Further, working memory is intrinsic and is responsible for storing and manipulating information needed during planning, composing, and revision processes. Our recent study (Ahmed et al., 2022a) using structural equation modeling (SEM) showed the NSVW can be deconstructed into key correlates (cognitive resources: self-efficacy and executive function), components (lower-order writing: handwriting and spelling; higher-order writing: planning, editing, and revising), and attributes of writing (productivity, quality, complexity, etc.) with multiple relations within and across the model. Similar to Ahmed et al. (2022a), the present study operationally defines the editing component of the NSVW as a broad construct that includes the knowledge of grammar, spelling, punctuation, and capitalization rules, as well as the ability to effectively apply these rules during the writing process. Likewise, in our study, we operationalized the concept of revision as a comprehensive construct that involves an understanding of syntax and structure, such as the development and organization of sentences and paragraphs, and the effective integration of this knowledge into the child’s written work. These definitions emphasize that the NSVW considers planning, editing, and revising as executive functions that necessitate the manipulation of information during the writing process beyond a mere understanding of grammar and syntax in oral language. The significance of these definitions lies in their recognition of the critical role played by higher-order cognitive processes in the writing process, such as the capacity to plan and organize ideas, pay attention to details, and revise written work for clarity and coherence. Consequently, the NSVW places equal emphasis on both declarative and procedural linguistic knowledge, highlighting the importance of not only understanding the rules of language but also applying them effectively in written expression.

### Joint models of reading-writing

An early component skills model linking R-W development is the Simple View of Reading and Writing (SVRW; Juel et al., 1986), which specified common predictors of word recognition and spelling (e.g., lexical knowledge) but did not find support for connections among spelling and word recognition or among reading comprehension and writing. In the SVRW, oral language and IQ were exogenous factors which indirectly influenced spelling through their effect on phonemic awareness (i.e., the effect of oral language on spelling was completely mediated by phonemic awareness). More recently, Kim (2020) developed the Interactive Dynamic Literacy Model (IDL) and the Direct and Indirect Effects Model of Writing (DIEW; Kim and Graham, 2022). The premise of the IDL and DIEW is that several related, yet separate, systems support R-W and include oral language, knowledge, domain-general and higher-order cognition, and sociocultural systems. The IDL model is situated within a levels of language framework, including discourse (text reading fluency, text writing or composition fluency), sentence level (sentence comprehension and sentence writing fluency), lexical (word reading fluency, spelling fluency), and sub-lexical (phoneme-grapheme correspondence, transcription fluency) levels. Interactive relations are highlighted in both models with R-W skills developing interdependently within and across a hierarchy. For example, reading comprehension influences composition and the experience of generating compositions can enhance comprehension through promoting awareness of structure and meaning of text. Reading comprehension is also expected to vary as a function of dimension of written composition (e.g., writing quality, productivity, correctness in writing, syntax, story structure, etc.; Shanahan and Lomax, 1986).

The IDL and DIEW models are broad frameworks that build on older R-W models described above. The empirically tested versions of the IDL and DIEW models are narrower in the number and types of components included and the associations among them. The models do not specify direct relations among higher-order R-W (e.g., vocabulary or inferencing to written expression) because the tested models postulated a complete mediation of higher-order skills through their effects on oral language. In the DIME model described above, higher-order skills (vocabulary, background knowledge, inferencing, and strategies) are pre-requisite reading skills, and collectively replace the linguistic comprehension component of the Simple View of Reading. In contrast, in the empirically tested IDL and DIEW, oral language plays a central role. The same higher-order skills (vocabulary, background knowledge, inferencing, and strategies) are specified as pre-requisite oral language skills, such that higher-order skills only influence reading comprehension (and writing) indirectly through their effect on oral language. Additional research is needed to better understand the nature of the indirect *and* direct relations between higher-order R-W processes to provide critical information that informs interventions for students with LDs.

## Current study

**Figure 1:**
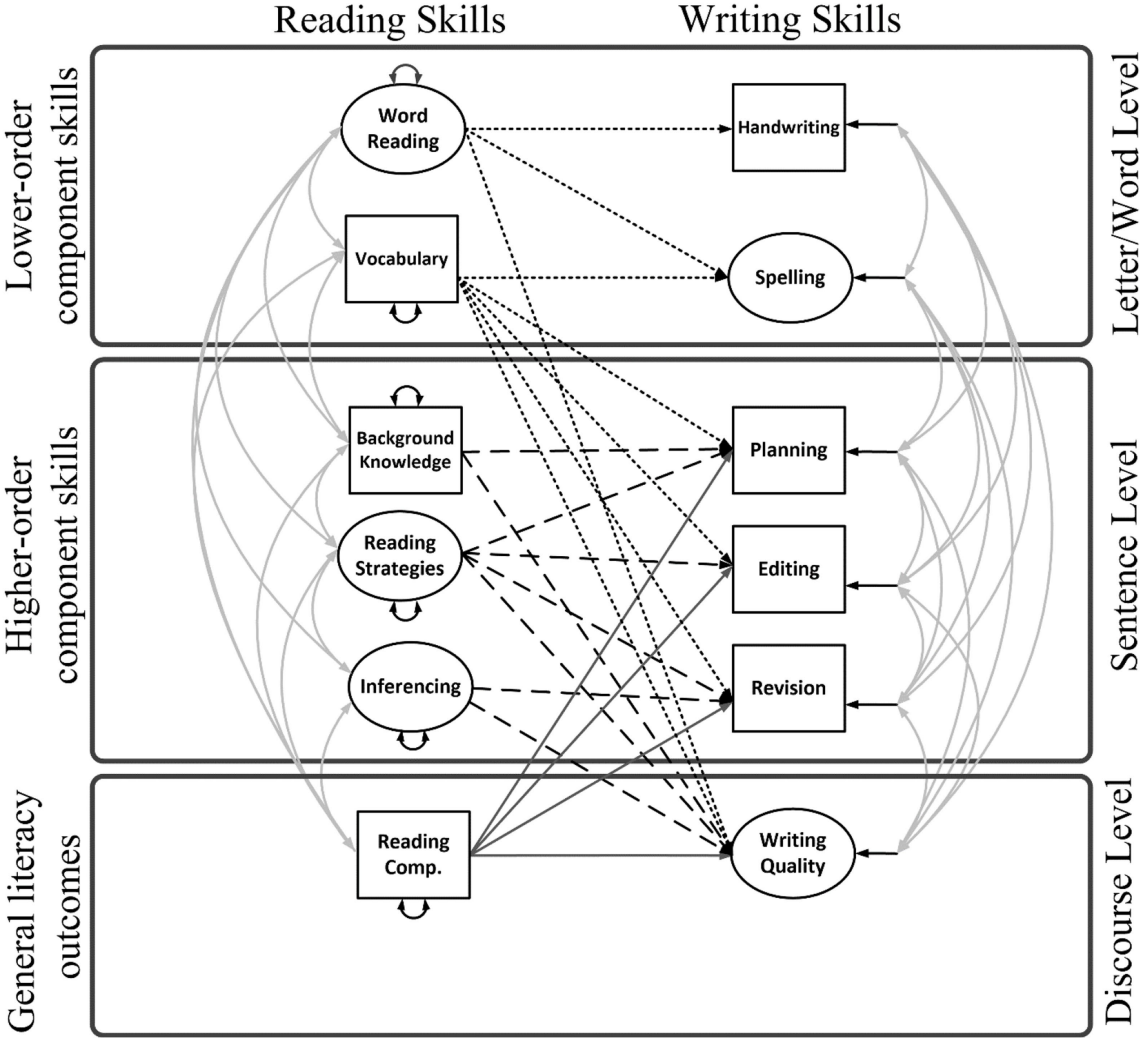
Reading-to-Writing Skills (RWS) Model without reading and writing fluency (Model 1). The component skills are grouped under (1) letter or word level, which encompasses code- and meaning-based skills mainly at the word level; (2) the sentence level, which encompasses meaning-making linguistics skills; and (3) general literacy outcomes at the discourse level. The component skills roughly correspond with the levels of languages specified in the figure because the granularity of a component skill may be dependent on a child’s ability (e.g., planning may consist of single words for some students and sentences for others), the nature of the task (e.g., editing may involve correcting words or sentences), or the nature of scoring (e.g., legibility of letters and words were both considered for scoring handwriting quality). Small-dashed lines are 8 paths from lower-order reading skills to writing skills; long-dashed lines are 8 paths from higher-order reading skills to writing skills; solid lines are 4 paths from reading outcomes to writing skills. Double headed arrows are correlations or variances. Small single-headed arrows are residual variances.

The purpose of this study is to examine the relationships between multiple component skills of R-W in a sample of children with LD, focusing on two areas of research that have received the least attention: (1) connections among higher-order component skills of R-W (e.g., inferencing and revision); (2) connections among reading fluency and higher-order R-W. Our central research question was: What are the direct (Aims 1 and 2) and mediated (Aims 3 and 4) relations among R-W skills in comprehensive component-skills models? With the above-mentioned theoretical models and existing evidence as our foundation for understanding component skills involved in the R-W connection, the following four aims guided this question:

Aim 1: To build and test a Reading-to-Writing Skills (RWS) model of literacy by joining the DIME and NSVW component skills (Model 1, Figure 1). As shown in Figure 1, this model evaluates direct effects only, with lower- and higher-order DIME skills on the left-hand side and lower- and higher-order NSVW skills on the right-hand side.

1.1. We hypothesized *word- and text-level connections* among word reading and transcription skill (spelling and handwriting), and reading comprehension and written expression (e.g., Juel et al., 1986; Berninger et al., 2002; Abbott et al., 2010). Although decoding contributes to writing quality and productivity (Connelly et al., 2006; Decker et al., 2016), we hypothesized that after controlling for higher-order skills, word reading would *not* relate to distal, higher-order writing processes or overall writing quality or productivity (Shanahan and Lomax, 1986; Morris et al., 2017), but vocabulary would significantly predict writing quality (e.g., Shanahan and Lomax, 1986; Olinghouse and Wilson, 2013; Allen et al., 2014; Carretti et al., 2016; von Koss Torkildsen et al., 2016; Neumann et al., 2020; Truckenmiller and Petscher, 2020).

1.2. We hypothesized *word-sentence level connections* of vocabulary with planning and editing (e.g., Vanderberg and Swanson, 2006) but not revision (von Koss Torkildsen et al., 2016).

1.3. We hypothesized *sentence-text level connections* among knowledge, inferencing, and reading strategies, and writing quality (Allen et al., 2014), along with direct connections among higher-order reading skills and composition processes gleaned from early theoretical models (e.g., Tierney and Pearson, 1983; Kucer, 1985): background knowledge with planning; reading strategies with planning, editing, and revision; and inferencing with revision. We hypothesized that inference would relate to revision rather than editing, which requires superficial changes to text compared to revision.

**Figure 2:**
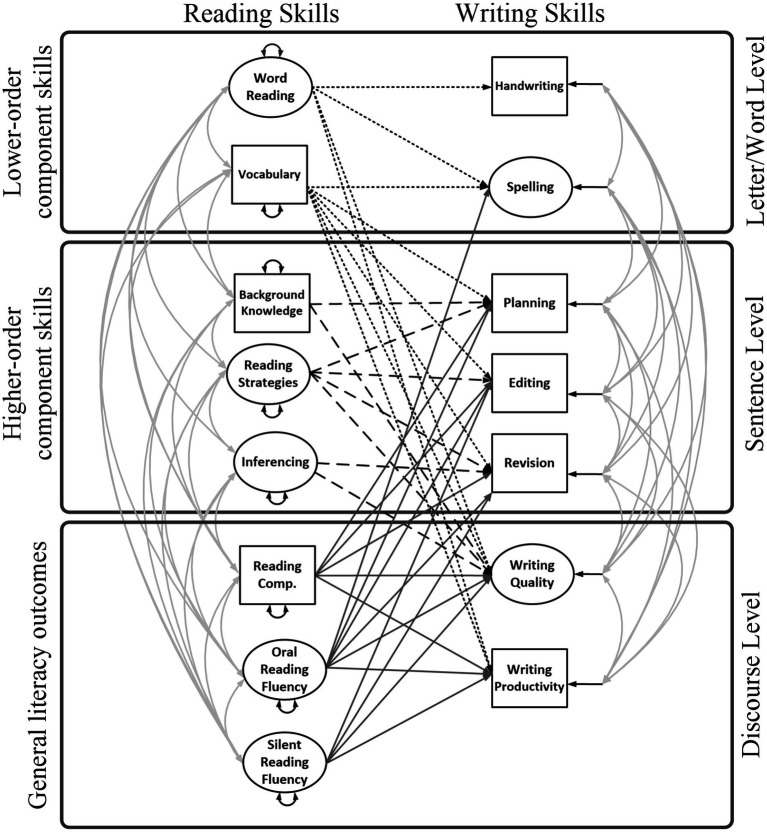
Full Reading-to-Writing Skills (RWS) Model with reading and writing fluency (Model 2). Silent reading fluency was measured using a sentence-level task, but it is included under discourse level because it measures comprehension of connected text and serves as a proxy for silent reading of longer texts. Small-dashed lines are 10 paths from lower-order reading skills to writing skills; long-dashed lines are 8 paths from higherorder reading skills to writing skills; solid lines are 15 paths from reading outcomes to writing skills. Double headed arrows are correlations or variances. Small single-headed arrows are residual variances.

Aim 2: To expand the Reading-to-Writing Skills model to include connections with R-W fluency (e.g., ORF and writing productivity; Model 2, Figure 2). As shown in Figure 2, we include ORF and SRF as measures of reading fluency and we incorporate writing productivity as the measure of discourse-level writing fluency.^1^

2.1. We hypothesized *word- and text-level connections* of vocabulary with writing productivity but not of word reading with productivity (von Koss Torkildsen et al., 2016). Furthermore, we hypothesized that for children with LDs ORF would relate to spelling (Bear, 1991; Lefly and Pennington, 1991), writing quality (Ritchey and Coker, 2014), and productivity (Ahmed et al., 2014; Tortorelli and Truckenmiller, 2023). We expected that after controlling for word-reading and ORF, SRF would not relate to word-level writing.

2.2. We hypothesized *sentence-text level connections* among reading fluency, writing processes (e.g., editing and revision). We expected that after controlling for ORF and SRF, reading comprehension would predict planning and writing quality but not productivity or self-regulatory processes of editing and revision, and that ORF would relate to planning (Bear, 1991) but not revision (von Koss Torkildsen et al., 2016), and SRF would relate to revision (Ahmed et al., 2014).

**Figure 3:**
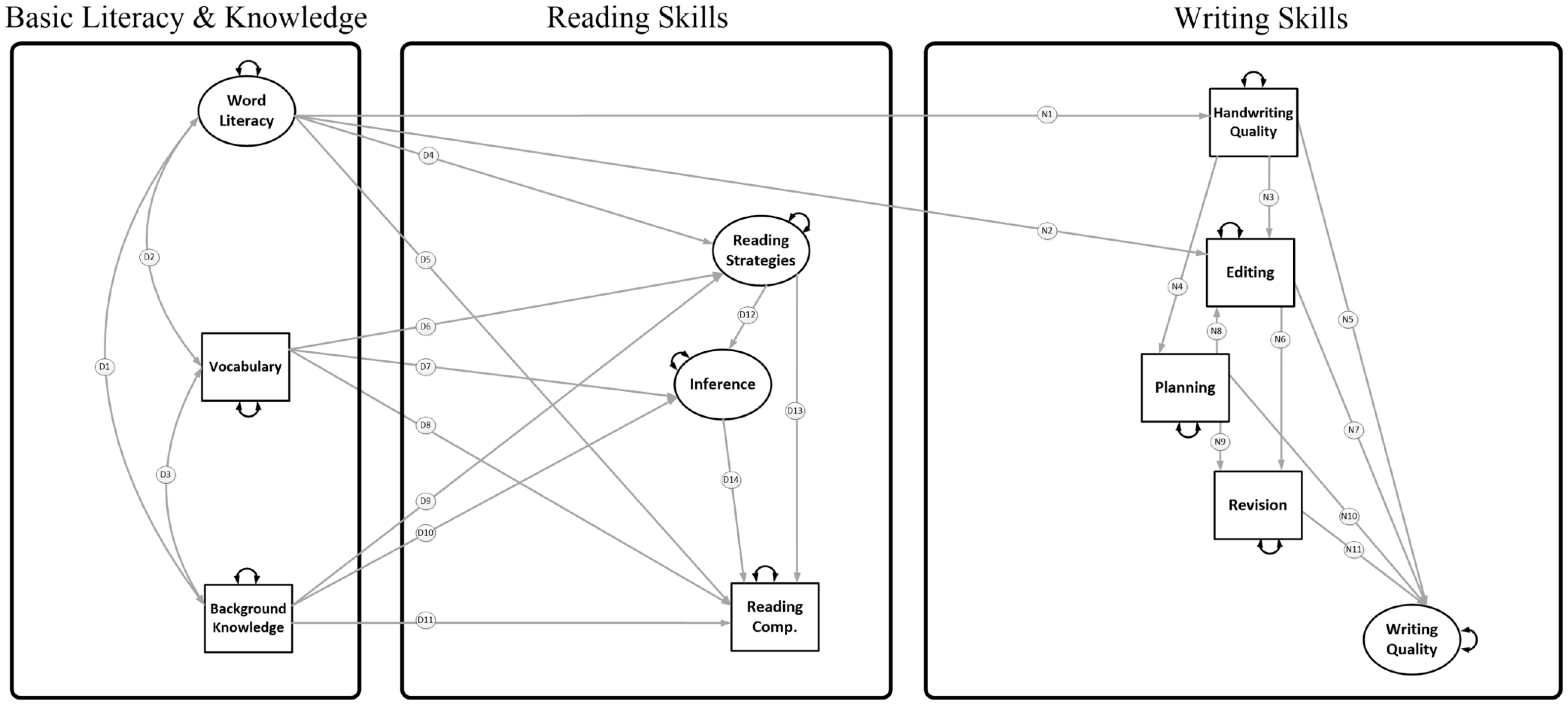
Reading-to-Writing Mediation (RWM) model (with cross-domain associations omitted for illustration; Model 3). The word literacy factor was measured by indicators of word reading (WJ-III Letter Word Identification and TOWRE Sight Word Efficiency) and spelling (WJ-III Spelling and percent words spelled correctly). Paths D1-D13 are correlations, direct, and indirect effects from the DIME model. D4 was added to account for the relation between spelling and summary writing (i.e., reading strategies). Paths N1-N11 are direct and indirect effects from the NSVW model.

Aim 3: To build and test a Reading-to-Writing Mediation (RWM; Figure 3) model with multiple direct and indirect paths between DIME and NSVW component skills (Model 3). Figure 3 depicts the domain-specific direct and indirect effects specified by the DIME or NSVW models (cross-domain associations are omitted for illustration purposes).

3.1. We hypothesized *connections among reading skills* such that reading comprehension would be related to vocabulary, knowledge, and inferencing, but reading strategies would not be a significant predictor of reading comprehension (Ahmed et al., 2016). Word literacy (i.e., word decoding and encoding) would relate to reading comprehension but also to reading strategies because this factor was measured using writing-for-reading tasks (i.e., summarizing), which are related to spelling (Bahr et al., 2020).

3.2. We hypothesized *connections among writing skills* such that word literacy would relate to handwriting quality and editing, but not distal, higher-order writing skills (planning and revision) and that direct and indirect effects among handwriting, planning, editing, revision, and writing quality would remain significant in the mediation model.

**Figure 4:**
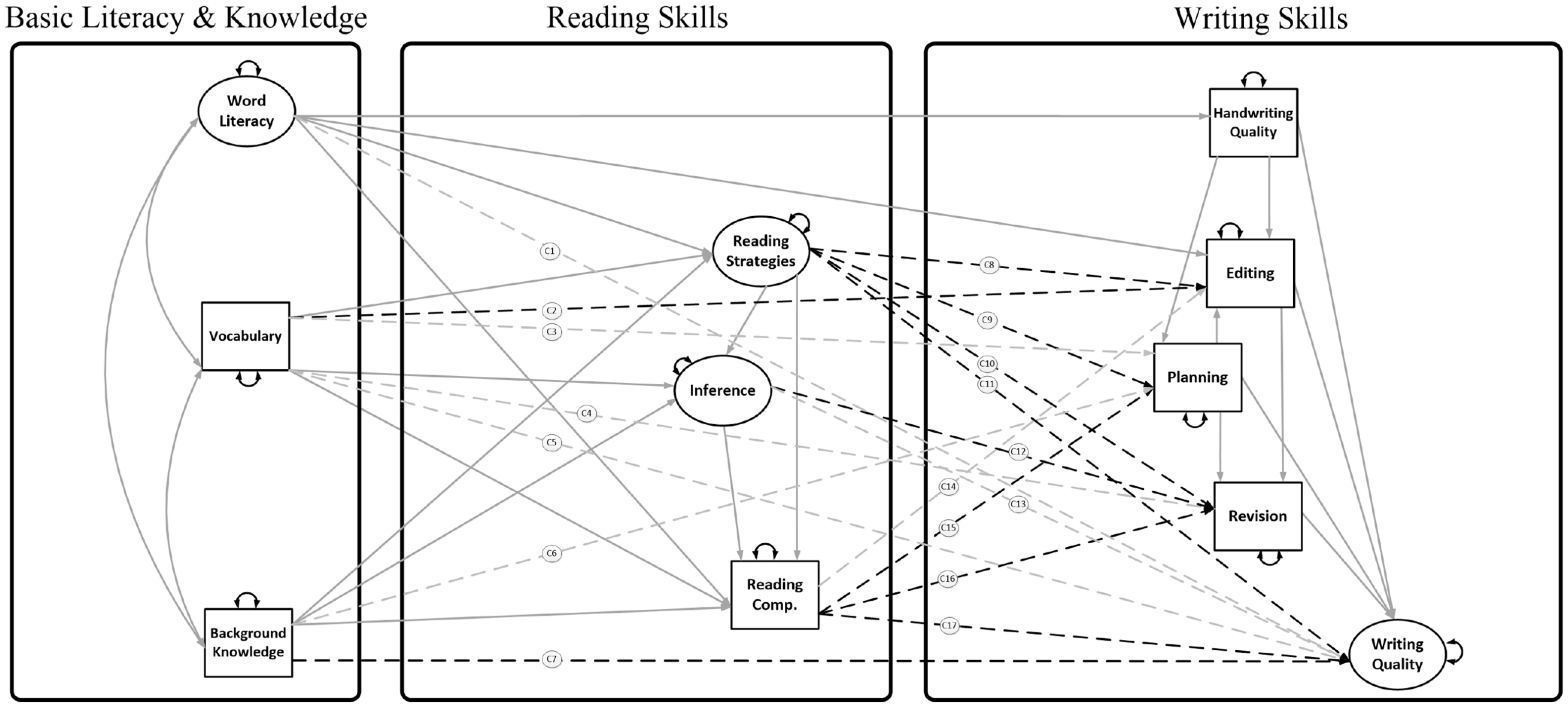
Reading-to-Writing Mediation (RWM) model (with cross-domain associations included; Model 3). Solid lines = within domain associations. Dashed lines = paths C1-C16 are cross-domain associations among reading and writing skills. Gray dashed lines were tested but omitted from the final model; black dashed lines were included in the final Model 3.

3.3. We hypothesized *cross-domain connections* among higher-order reading (vocabulary, knowledge, inferencing, and strategies) and higher-order writing (planning, editing, revision) as well as reading comprehension and written expression. Figure 4 shows a mediation model in which (a) higher-order reading mediated the relations of basic literacy and knowledge with higher-order writing (e.g., vocabulary ➔ inferencing ➔ revision), (b) higher-order writing mediated the relations of higher-order reading and writing quality (e.g., reading strategies ➔ editing ➔ writing quality), and (c) higher order writing mediated the relations of basic literacy and knowledge with writing quality (e.g., background knowledge ➔ planning ➔ writing quality).

**Figure 5:**
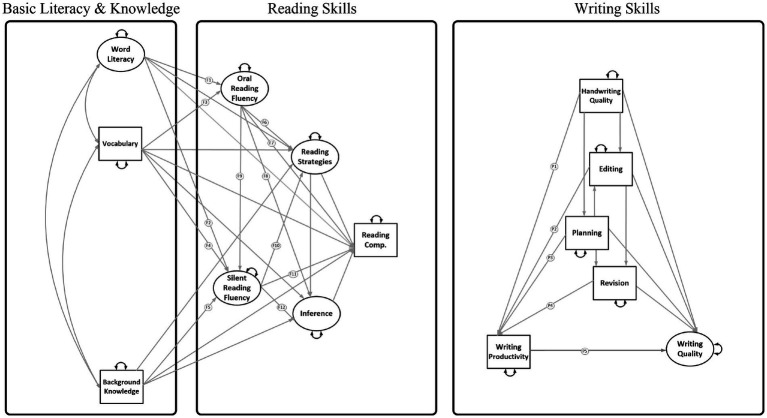
Full Reading-to-Writing Mediation (RWM) model (with cross-domain associations omitted for illustration; Model 4). Paths F1-F12 are paths to/from oral or silent reading Fluency; paths P1-P5 are paths to/from writing Productivity (fluency).

Aim 4: To expand the Reading-to-Writing Mediation model to include direct and indirect connections with R-W fluency (e.g., ORF, writing productivity; Model 4). Figure 5 depicts the domain-specific direct and indirect effects after including oral and silent reading fluency and writing productivity (cross-domain associations are omitted for illustration purposes).

4.1. We hypothesized *connections among reading skills* such that the direct effects of basic literacy and knowledge on reading comprehension would no longer be significant, but indirect effects would be found via oral and silent reading fluency. We hypothesized that connections among higher-order DIME skills would also change after controlling for SRF and ORF.

4.2. We hypothesized *connections among writing skills* such the direct and indirect effects of writing skills would be significant for writing productivity and would remain significant for writing quality.

**Figure 6:**
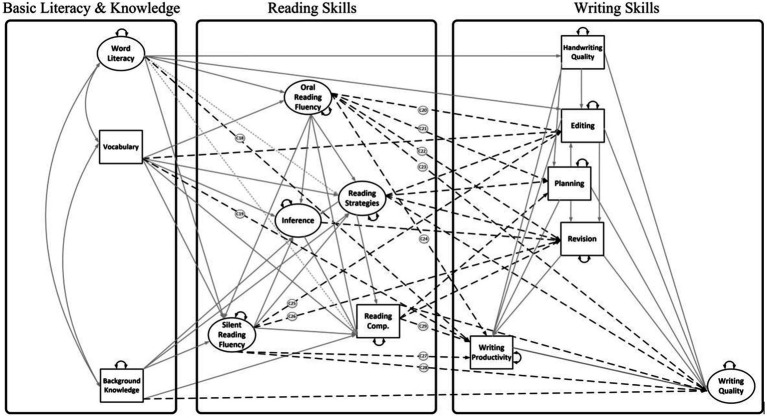
Full Reading-to-Writing Mediation (RWM) model (with cross-domain associations included; Model 4). Within-domain variables and paths are the same as Figure 5 but were rearranged to include cross-domain paths. Long-dashed lines = paths C17-C28 are cross domain associations to/from oral and silent fluency and writing productivity. Solid lines = within domain associations. Small-dashed lines = the model did not converge with the inclusion of these paths.

4.3. We hypothesized *cross-domain connections* would change as a function of R-W fluency. Figure 6 shows a mediation model in which higher-order reading mediated the relations of ORF and SRF with writing skills, and higher-order writing mediated the relations of ORF and SRF and writing quality because we hypothesized that fluent reading foments deeper cognitive processing (e.g., reading comprehension and revision), which in turn influence writing quality and productivity (e.g., silent reading fluency ➔ inferencing ➔ revision; oral reading fluency ➔ editing ➔ writing quality). In general, we expected SRF to mediate the effects of ORF on other R-W skills, noting that the literature on indirect effects is limited.

## Method

### Participants and procedures

Data were collected as part of a larger RCT of an after-school reading intervention (see Roberts et al., 2018) with a 2×2 factorial treatment design with 2 levels of reading intervention (foundational reading skills and text-processing or text-processing only) and 2 levels of modality of small group instruction (writing or self-regulation). All intervention conditions included individualized computer-based instruction and small-group instruction in the first and second phase, respectively, of each instructional session. However, the present study includes data from pre-test only (i.e., the interventions took place after the collection of the baseline battery of measures included in the present study and did not impact performance on any tests). Thus, in this study, we do not differentiate among experimental condition because no differences were apparent in the groups randomized to treatment and the business-as-usual control condition on the assessments or on demographic variables. The sample for the present study consisted of 405 children in Grades 3–5 who were identified as struggling readers using the 25th percentile cutoff on the Test of Silent Reading Efficiency and Comprehension (TOSREC). These students also struggled with written expression as evidenced by low scores on the Test of Written Language (TOWL). As Table 1 indicates, the average age of the sample was 10 and ranged from 6 to 12 years old. Most of the sample was economically disadvantaged (69% free/reduced lunch) and 20% were in special education and/or had limited English proficiency. The majority of the sample was White (52%), followed by Black (41%), multiple races (22%), American Indian or Alaskan Native (1.5%), and Asian (0.5%).

**Table 1:** Demographic characteristics by grade level.

Variable	Grade 3	Grade 4	Grade 5	Total
*n*	114	152	139	405
Age mean (SD)	8.81 (0.58)	9.77 (0.50)	10.84 (0.54)	9.85 (0.96)
Age range	6–10	7–11	9–12	6–12
Female	56 (52%)	81 (57%)	70 (52%)	207 (54%)
Free/Reduced lunch	89 (78%)	99 (65%)	92 (66%)	280 (69%)
Limited English proficiency	23 (28%)	20 (16%)	22 (19%)	65 (20%)
Special education	17 (20%)	16 (13%)	32 (28%)	65 (20%)
Race				
African American or Black	48 (45%)	52 (37%)	55 (41%)	155 (41%)
American Indian and Alaska native	1 (<1%)	3 (2%)	2 (2%)	6 (1.5%)
Asian	2 (2%)	–	–	2 (0.5%)
Multiple races	4 (4%)	11 (8%)	7 (5%)	22 (6%)
White	52 (49%)	76 (54%)	70 (52%)	198 (52%)

## Measures

### Reading measures

#### Word reading

Word reading was assessed using the Woodcock Johnson III Letter Word Identification (WJ-LWID; Woodcock et al., 2001) and the Sight Word Efficiency (SWE) subtest of the Test of Word Reading Efficiency (TOWRE; Torgesen et al., 1999), with both measures demonstrating adequate reliability (*α* = 0.91 and *α* = 0.90–0.93, respectively).

#### Vocabulary

The verbal knowledge subtest of the Kaufman Brief Intelligence Test (KBIT-2; Kaufman, 2004) was used to measure picture vocabulary. The students are required to point to a picture that shows the meaning of a word or provides the answer to a question. Reliability is adequate for verbal knowledge subtest (*α* = 0.86–93).

#### Inferencing

The Bridge-It (Pike et al., 2010) test of inferencing measures the ability to integrate information presented in a statement sentence and a continuation sentence. Students are asked to read 4 sentences, one of which is the statement sentence, and a continuation sentence, which can either be a correct continuation (i.e., consistent with the situation model) or an incorrect continuation (i.e., inconsistent with the situation model). The statement sentence and the continuation sentence were separated by 3 sentences in the far condition and were adjacent in the near condition. This measure has adequate reliability (*α* = 0.73).

#### Background knowledge

The Assessment of Writing, Self-Monitoring and Reading (AWSM Reading; Gioia et al., 2023) is a paper-and-pencil experimental test developed for the larger study. Background knowledge items were tied directly to three passages that students read for comprehension and were not tied to the topic the students wrote about. The background knowledge items (e.g., *What is found inside Yellowstone National Park?*) were presented prior to reading the passages. A composite score of the knowledge items was used in this study (*α* = 0.62–0.69).

#### Reading strategies

Items from the AWSM Reader were also used to form a latent variable for strategies. Students read passages and provided short summaries of the passages as a performance measure of reading strategies. Reliability was high for summary writing (*κ* = 0.92–0.97) in this sample. The strategies factor also included a self-report measure of contextualized learning, Student Contextual Learning Scale (SCL; Cirino, 2012). The Strategies sub-scale of the SCL asks students to rate their beliefs, attitudes, and habits related to reading and learning strategies, with adequate reliability (*α* = 0.71–0.82) in our sample.

#### Reading comprehension

The Gates–MacGinitie Reading Test (GMRT; MacGinitie et al., 2000) was used to measure reading comprehension. The GMRT requires students to read short passages and answer multiple choice questions. The test has adequate reliability (Kuder–Richardson 20 [K-R 20] = 0.93–0.94 for grades 3–5).

#### Oral reading fluency

Two forms of the AIMSweb Oral Reading Fluency (Shinn and Shinn, 2002) were administered. Students were asked to read appropriate grad-level passages and the number of words read correctly in 1 min were recorded. AIMSweb reports adequate alternate-forms reliability (*α* = 0.80–0.81).

#### Silent reading fluency

Two forms of the Test of Silent Reading Efficiency and Comprehension (TOSREC; Wagner et al., 2010) were used. The TOSREC requires students to read sentences and verify the veracity of sentences (e.g., *Do birds fly?*). Alternate-forms reliability is high (0.86–0.93) in grades 3–5.

### Writing measures

#### Spelling

The Woodcock Johnson III (WJ-III; Mather et al., 2001) Spelling subtest required students to spell phonetically regular (e.g., *under*) and irregular (e.g., *beautiful*) words. Reliability is high for grades 3–5 (*α* = 0.93). Spelling was also measured by counting the percent of total words spelled correctly on the TOWL Story Composition subtest (described below), with high inter-rater reliability (*κ* = 0.98).

#### Handwriting

Scores were derived using the presentation domain (and handwriting subdomain) of the 6 + 1 traits rubric (described below). Inter-rater reliability was high (*κ* = 0.91).

#### Planning

Students were given 5 min to plan their TOWL Story Composition responses following TOWL administration guidelines (Hammill and Larsen, 2009). Because the TOWL does not include a separate rubric for scoring planning, we adapted a planning rubric from Olinghouse and Graham (2009), which consists of scores ranging from 1 (little or no planning) to 5 (detailed story elements). Inter-rater reliability was *κ* = 0.75.

#### Editing and revision

Items for the editing and revision measures were derived from the TOWL Contextual Conventions subscale. Editing items required knowledge of mechanics and Revising items required knowledge of writing elements that enhance meaning. Two independent raters classified the 21 items into two categories (editing or revision), with perfect agreement (*κ* = 1.00). The editing category included items related to grammar, capitalization, spelling, and punctuation. The revision category included items related to content, structure, syntax and organization (see Supplementary Appendix C for the list of items coded as editing or revision). Internal consistency in this sample was *α* = 0.62 for Editing and *α* = 0.72 for Revision. As additional evidence for internal validity, we present a factor model for the TOWL Editing and Revision sub-scales as supplementary analyses in Supplementary Appendix C. Further, external validity was established with the State of Texas Assessments of Academic Readiness (STAAR; Texas Education Agency, 2016) Editing and Revising subtests with a sub-sample of fourth grade students (*n* = 73) from the present study for whom data were available on the state-wide assessment. The STAAR test was administered in the semester following the administration of the TOWL. Data were only available for a subsample of students because the STAAR high stakes writing assessment is not administered in grades 3 or 5 in Texas and because the STAAR Writing data were obtained for a smaller project (see Reid et al., in press). The STAAR Editing and Revision subtests require reading grade-level compositions embedded with errors and answering multiple-choice questions to identify and/or correct the errors in the text (see Reid et al., in press, for additional details). The STAAR and TOWL Editing sub-scales (*r* = 0.51, *p* < 0.001) and the STAAR and TOWL Revision sub-scales (*r* = 0.43, *p* < 0.001) were moderately correlated.

#### Writing quality

The Story Composition subtest of the Test of Written Language – 4th Edition (TOWL; Hammill and Larsen, 2009) requires students to write a story in response to a picture prompt in 15 min. We used the TOWL scoring guidelines to obtain the Story Composition score, which is scored on criteria such as plot (storyline), if characters show feelings/emotions, and story action or energy level. In addition, we used the 6 + 1 traits rubric (Culham, 2003) to score the essays. The 6 + 1 traits rubric includes the following domains: (1) Ideas: whether the essay is focused and clearly communicates ideas; (2) Organization: if the logical structure makes ideas easy to follow; (3) Voice: whether the author writes in an engaging manner; (4) Word Choice: relates to how the student’s choice of words creates a clear vision for the reader; (5) Sentence Fluency: how the author uses sentences and phrases to communicate; and (6) Conventions: errors related to punctuation, spelling, capitalization, grammar/usage. Inter-rater reliability ranged from *κ* = 0.80 for ideas to *κ* = 0.91 for word choice.

#### Writing productivity

The total words written were obtained for the TOWL Story Composition responses, as were correct minus incorrect word sequences (CIWS). CIWS is a curriculum-based measure of written grammar and mechanics (Espin et al., 2008). If two adjacent words are correctly spelled, capitalized, and punctuated that bigram results in a correct word sequence; otherwise, the bigram results in an incorrect word sequence. CIWS is calculated as the correct sequences minus any incorrect word sequences. Inter-rater reliability was *κ* = 0.995 for TWW and *κ* = 0.98 for CIWS.

### Analytic approach

The present study consists of a secondary data analysis employing SEM to separately evaluate relations *among* reading components using the DIME model of reading comprehension as a theoretical framework (Ahmed et al., 2022b) and writing components using the NSVW as the theoretical framework (Ahmed et al., 2022a). We evaluate the relations *between* R-W components from these models, as shown in Figures 1, 2, 4, 6. The lack of empirical support for a path may reflect that research is lacking in a specific area, rather than support for a null relationship. Therefore, several paths were evaluated that have theoretical support but little empirical support (e.g., reading fluency and writing). Paths were not estimated if they were not significant in prior studies and there was no support in the theoretical literature (e.g., inference to handwriting and inference to spelling; Kim, 2020).

The SEM models were fit using Full Information Maximum Likelihood (FIML) in M-plus 8.6 (Muthen and Muthen, 1998–2017) to handle missing data (in the current sample, covariance coverage ranged from 0.81 to 0.99). Multiple criteria were considered to evaluate a model fit function (i.e., the extent to which the model fits the data) given a specific estimation method. Absolute model fit was evaluated using Akaike Information Criteria (AIC), Bayesian Information Criteria (BIC) and sample-size adjusted BIC, which take sample size, model fit, and number of parameters into account, with lower values reflecting a better fit. The root mean square error of approximation (RMSEA) compensates for model complexity and standardized root mean square residual (SRMR) is the standardized difference between the observed and predicted correlations. RMSEA and SRMR values ≤0.05 indicate an adequate fit. The comparative fit index (CFI) and Tucker-Lewis index (TLI) are incremental indices that compare the fit of the hypothesized model with a more restricted, baseline model (i.e., a model in which all observed variables are uncorrelated). CFI and TLI values ≥0.95 indicate a good fit and values ≥0.90 indicate an acceptable fit (Hu and Bentler, 1999).

#### Aims 1 and 2 (direct effects models)

Aim 1 examined the direct effects of DIME to NSVW component skills shown in Figure 1 (Model 1). Aim 2 examined the direct effects of R-W skills with three nested models that systematically incorporated R-W fluency: a full^2^ R-to-W Skills model depicted in Figure 2, and two nested models in which relations of each fluency skill (ORF or SRF) with writing skills were estimated independently of the other fluency skill. For example, the ORF model excluded any hypothesized relations of SRF with writing skills. The SRF model included all paths from the full model (Figure 2) but excluded any relations of ORF with writing skills. Nested models were compared with chi-square difference tests.

#### Aims 3 and 4 (mediation models)

The RWM models explored indirect effects of basic literacy (i.e., decoding and encoding) and knowledge (i.e., word and world knowledge) on writing skills via the indirect effects on reading skills. In addition, several indirect effects were evaluated within the reading domain (e.g., vocabulary ➔ inference ➔ reading comprehension) and writing domain (e.g., handwriting ➔ editing ➔ writing quality). The measurement models were similar to the RWS models of Aims 1–2, except that measures of word reading and spelling loaded on a single factor (Mehta et al., 2005). Consequently, in the RWM models, the word literacy factor predicted multiple R-W skills. As a first step, the RWM models included associations among DIME skills (paths D1-D14 in Figure 3) and NSVW skills (paths N1-N11 in Figure 3). We then evaluated the cross-domain associations shown in Figure 4 (paths C1-C17) by testing competing structural models. Due to the specification of a word literacy factor, these cross-domain associations are the 17 paths from Model 1 (Figure 1) which did not involve handwriting or spelling. The RWM model without fluency (Model 3) was generated by trimming paths without strong empirical support (i.e., gray dashed arrows in Figure 4; Mulaik and Millsap, 2000). The full RWM model with fluency (Model 4) retained the same variables and paths from the trimmed Model 3 and incorporated three R-W fluency variables (ORF, SRF, and writing productivity; Figure 5). Several direct and indirect effects were evaluated within the reading domain (e.g., ORF ➔ SRF ➔ inference ➔ reading comprehension; paths F1-F12 in Figure 5) and writing domain (e.g., handwriting ➔ productivity ➔ quality; paths P1-P5 in Figure 5) based on the literature reviewed. Cross-domain associations from Model 3 were retained (including ten cross-domain paths C2, C8-C12, C15-C17 which were not trimmed), and additional cross-domain associations were evaluated with R-W fluency skills (paths C18-C29 in Figure 6). The additional cross-domain associations are the 12 paths from Model 2 (Figure 2) originating from ORF or SRF, or going into writing productivity (with the exception of ORF ➔ spelling because in the RWM models spelling was combined with word reading in the word literacy factor). All indirect effects were estimated under FIML in Mplus and bias-corrected bootstrapped confidence intervals were obtained based on 1000–3000 bootstrap samples.

#### Alternative direct effects models

For space considerations the diagrams and results for the alternative models are presented in the Appendices. First, the Reading-to-Writing Domains model explored how reading skills differentially relate to writing *dimensions* depending on the skills assessed (Shanahan and Lomax, 1986; Kim and Graham, 2022). For example, ORF may predict sentence fluency because this dimension taps into the ability to use varied sentence structures that invite expressive oral reading. Similarly, ORF may predict scores on the voice dimension because this dimension taps into the ability to address the reader in an engaging way. In the present study, we measured six dimensions using the 6 + 1 traits rubric (Culham, 2003). In our approach to examining the R-W relationship, we also use correct minus incorrect word sequences (CIWS) as an overall indicator of writing. As a production-dependent metric, CIWS captures the amount of written text a student produces but also captures writing quality through consideration of spelling, grammar, and punctuation of adjacent words in the context of a sentence. Three R-to-W Dimensions models were evaluated: a full model depicted in Supplementary Appendix A and two reduced models for ORF and SRF, respectively. The R-to-W Dimensions Model included a general factor for writing which reflects the common variance across specific writing dimensions. Thus, the path from a specific reading skill to a specific dimension can be interpreted as a one unit change in the writing dimension as a function of the reading skill after controlling for (a) other reading skills and (b) for variance shared with other dimensions. Second, we evaluated a Writing-to-Reading Model because it is possible that the opposite directionality could fit the data equally well (i.e., due to model equivalence). Like the R-to-W models, paths for the W-to-R models were specified based on prior literature. For example, if there were no theoretical, experimental, or correlational studies surmising that better planning influences vocabulary then this path was omitted from the model. The W-to-R model (see Supplementary Appendix B) specified that: (a) handwriting and spelling predicted word reading; (b) spelling also predicted vocabulary, knowledge, ORF, and SRF; (c) higher-order writing (planning, editing, and revision) predicted reading strategies and inferencing; (d) planning predicted reading comprehension (e) editing and revision predicted reading comprehension, ORF, and SRF; (f) writing productivity predicted reading comprehension, ORF, and SRF; and (g) writing quality predicted all reading skills.

## Results

Data were first screened for assumptions of normality and outliers, defined as data points with studentized residuals ±3 and high leverage. As the outliers did not represent minor or major reliability concerns (e.g., equipment failure), and the inclusion of the outliers did not change the results substantively, these data points were retained for the final analyses. As shown in Table 2, all assumptions of univariate normality were supported. The higher kurtosis on the AWSM Reader Summary 3 (5.43) is due to this passage’s higher text difficulty (readability) in comparison to summary 1 and 2 (Gioia et al., 2023).

**Table 2:** Descriptive statistics.

Variable	*n*	Mean	SD	Min	Max	Skew	Kurtosis
*Word reading*							
TOWRE SWE^1^	365	85.04	12.90	55.00	127.00	−0.05	0.04
WJ-III LWID^1^	386	94.03	11.82	41.00	155.00	−0.05	3.12
*Vocabulary*							
K-BIT	387	49.21	11.86	4.00	77.00	−0.75	1.30
*Background knowledge*							
AWSM reader – background knowledge	378	2.27	0.82	0.00	3.00	−0.79	−0.33
*Strategies*							
AWSM reader – summary 1	349	0.95	1.17	0.00	6.00	1.23	1.00
AWSM reader – summary 2	343	0.99	1.20	0.00	5.00	1.06	0.35
AWSM reader – summary 3	333	0.41	0.69	0.00	4.00	2.05	5.43
SCLC - Strategies	387	17.39	5.08	4.00	27.00	−0.33	−0.41
*Inferencing*							
Bridge-it near condition	376	5.54	2.16	0.00	10.00	−0.06	−0.72
Bridge-it far condition	376	4.51	1.86	0.00	10.00	0.06	−0.37
*Reading comprehension*							
GMRT^1^	387	451.01	31.95	349.00	547.00	−0.23	0.08
*Oral reading fluency*							
AIMSweb 1	386	80.57	32.67	4.00	181.00	0.00	−0.20
AIMSweb 2	385	78.90	31.10	1.00	175.00	−0.02	0.03
*Silent reading fluency*							
TOSREC 1	405	13.56	5.28	0.00	26.00	−0.35	−0.09
TOSREC 2	405	13.76	5.56	0.00	27.00	−0.42	−0.07
*Handwriting*							
Handwriting quality	377	2.85	1.13	1.00	6.00	0.31	−0.27
*Spelling*							
WJ-III spelling^1^	385	91.48	13.36	40.50	122.00	−1.22	1.59
PWSC	356	84.12	11.06	44.31	100.00	−0.69	1.22
*Planning*							
Planning	377	1.57	0.79	0.00	4.00	1.26	1.08
*Editing*							
Editing	359	5.56	2.91	0.00	14.00	0.47	−0.31
*Revision*							
Revision	360	3.50	2.66	0.00	13.00	1.01	0.94
*Writing scores*							
TWW	371	96.75	43.35	13.00	251.00	0.35	−0.21
CIWS	356	37.41	43.42	−99.00^2^	207.00	0.24	0.18
Ideas	377	2.87	1.07	1.00	6.00	0.23	−0.24
Organization	377	2.78	1.13	1.00	5.00	0.11	−0.77
Voice	377	2.77	1.17	1.00	6.00	0.35	−0.45
Word choice	377	2.66	0.98	1.00	5.00	0.15	−0.21
Sentence fluency	377	2.10	1.07	1.00	5.00	0.77	−0.05
Conventions	377	2.19	0.89	1.00	5.00	0.37	−0.14
6 Traits total score	377	15.37	5.40	6.00	31.00	0.27	−0.27

^1^Standard score; all other scores are raw scores. ^2^Negative values indicate more incorrect word sequences than correct word sequences. CIWS, correct minus incorrect word sequences; PWSC, percent of total words spelled correctly; TWW, total words written.

### Reading-to-Writing Skills models

The reduced model depicted in Figure 1 (χ^2^ (108) = 215.96, *p* < 0.001; RMSEA [90% CI] = 0.05 [0.04, 0.06]; CFI = 0.96; TLI = 0.93; SRMR = 0.04) and the full model depicted in Figure 2 (χ^2^ (177) = 294.58, *p* < 0.001; RMSEA [90% CI] = 0.04 [0.03, 0.05]; CFI = 0.97; TLI = 0.95; SRMR = 0.04) provided a good fit to the data. These models explained 66–68% of variance in writing quality, 57–63% in spelling, 41–42% in editing, and a smaller percentage of variance in revision (22%), handwriting (12–16%), and planning (10%). The full RWS model also explained 16% variance in writing productivity (see Table 3).

**Table 3:** Variance explained in the direct effects models (RWS), mediation models (RWM), and alternative models.

Model	Handwriting	Spelling	Word literacy	Planning	Editing	Revision	Writing productivity	Writing quality
RWS Model 1	0.16	0.63	N/A	0.10	0.42	0.22	N/A	0.68
RWS Model 2	0.12	0.57	N/A	0.10	0.41	0.22	0.14	0.66
RWM Model 3	0.19	N/A	N/A	0.13	0.60	0.32	N/A	0.84
RWM Model 4	0.16	N/A	N/A	0.14	0.79	0.32	0.23	0.90
	**Word reading**	**Vocabulary**	**Background knowledge**	**Reading strategies**	**Inferencing**	**Oral reading fluency**	**Silent reading fluency**	**Reading comprehension**
RWM Model 3	N/A	N/A	N/A	0.50	0.50	N/A	N/A	0.49
RWM Model 4	N/A	N/A	N/A	0.51	0.62	0.90	0.93	0.52
W-to-R skills	0.51	0.16	0.21	0.51	0.45	0.56	0.58	0.42
	**Organization**	**Voice**	**Word choice**	**Sentence fluency**	**Conventions**	**Ideas**	**CIWS**	
R-to-W domains	0.79	0.76	0.68	0.52	0.51	0.83	0.42	

CIWS, Correct minus incorrect word sequences; RWS, Reading-to-Writing Skills; RWM, Reading-to-Writing Mediation; W-to-R, Writing to Reading. N/A, variable was an exogenous variable (word literacy, word reading, vocabulary, background knowledge) or was not included in the model (spelling, word literacy, oral/silent reading fluency or total words written).

All measures loaded significantly on their hypothesized factors (see Table 4). For the reading strategies factor, the loading of the self-report measure was smaller in magnitude (*λ*=0.13–0.14, *p* < 0.05) because all other loadings on this factor were from performance measures of strategies (summarizing). The correlations among reading variables in the full model are reported in Table 5, and correlations among residuals of the writing variables are reported in Table 6. Most reading variables were moderately to highly correlated, ranging from 0.25 for word reading and vocabulary (and reading strategies and vocabulary) to 0.90 for ORF and decoding. The largest residual correlation for the writing variables was between revision and writing quality (*r* = 0.63), and the smallest correlation was between spelling and total words written (*r* = 0.03; see Table 6). In addition, the disturbances of the WJ spelling and word reading subtests were allowed to correlate in all models because both subtests belong to the same family of tests (*r* = 0.46–0.54, *p* < 0.05).

**Table 4:** Standardized solutions for the measurement models.

Variable	Reduced R-to-W skills (Model 1)		Full R-to-W skills (Model 2)			Reduced R-to-W mediation (Model 3)		Full R-to-W mediation (Model 4)	
	Parameter	SE	Parameter	SE	Variable	Parameter	SE	Parameter	SE
*Word reading*					*Word literacy*				
TOWRE SWE	0.69**	0.04	0.82**	0.03	TOWRE SWE	0.59**	0.05	0.71**	0.04
WJ LWID	0.70**	0.04	0.70**	0.03	WJ LWID	0.59**	0.05	0.62**	0.04
*Spelling*					WJ spelling	0.79**	0.04	0.70**	0.03
WJ spelling	0.87**	0.02	0.87**	0.02	%WSC	0.78**	0.04	0.68**	0.03
%WSC	0.84**	0.03	0.83**	0.02					
*Reading strategies*					*Reading strategies*				
CLS: strategies	0.14*	0.06	0.13*	0.06	CLS: strategies	0.14*	0.06	0.14*	0.06
Summary 1	0.69**	0.04	0.71**	0.04	Summary 1	0.71**	0.04	0.72**	0.04
Summary 2	0.83**	0.03	0.82**	0.03	Summary 2	0.81**	0.03	0.80**	0.04
Summary 3	0.65**	0.04	0.69**	0.04	Summary 3	0.68**	0.05	0.68**	0.05
*Inference*					*Inference*				
Bridge-It Near	0.78**	0.05	0.77**	0.05	Bridge-It Near	0.77**	0.05	0.77**	0.05
Bridge-It Far	0.53**	0.05	0.53**	0.05	Bridge-It Far	0.54**	0.05	0.54**	0.05
*Oral reading fluency*					*Oral reading fluency*				
AIMSweb 1	N/A	N/A	0.92**	0.01	AIMSweb 1	N/A	N/A	0.92**	0.01
AIMSweb 2	N/A	N/A	0.91**	0.01	AIMSweb 2	N/A	N/A	0.91**	0.01
*Silent reading fluency*					*Silent reading fluency*				
TOSREC 1	N/A	N/A	0.72**	0.03	TOSREC 1	N/A	N/A	0.67**	0.04
TOSREC 2	N/A	N/A	0.73**	0.03	TOSREC 2	N/A	N/A	0.69**	0.04
*Writing*					*Writing*				
TOWL story composition	0.73**	0.03	0.74**	0.03	TOWL story composition	0.73**	0.03	0.75**	0.03
6 + 1 traits	0.81**	0.03	0.80**	0.03	6 + 1 traits	0.82**	0.03	0.80**	0.03

**p < 0.001, *p < 0.05.

N/A, The variable was not included in the reduced models.

**Table 5:** Correlations among exogenous variables in the full Reading-to-Writing Skills model (Model 2).

	WORD	VOC	BK	RS	INF	RC	ORF
WORD	–						
VOC	0.25	–					
BK	0.34	0.32	–				
RS	0.58	0.25	0.39	–			
INF	0.50	0.42	0.42	0.60	–		
RC	0.38	0.37	0.44	0.48	0.65	–	
ORF	0.90	0.53	0.44	0.56	0.53	0.60	–
SRF	0.73	0.33	0.57	0.59	0.68	0.65	0.81

All correlations are significant at *p* < 0.001. WORD, word reading; BK, background knowledge; RS, reading strategies; INF, inferencing; RC, reading comprehension; ORF, oral reading fluency; SRF, silent reading fluency.

**Table 6:** Correlations among disturbances of writing variables from the full Reading-to-Writing Skills model (Model 2).

	HW	SPELL	PLAN	EDIT	REV	WQ
HW	–					
SPELL	0.12	–				
PLAN	0.18	0.06	–			
EDIT	0.24	0.44	0.10	–		
REV	0.15	0.31	0.16	0.30	–	
WQ	0.52	0.35	0.33	0.29	0.63	–
TWW	0.21	0.03	0.18	0.06	0.22	0.50

Correlations above 0.10 are significant at *p* < 0.05. HW, handwriting; SPELL, spelling; PLAN, planning; EDIT, editing; REV, revision; WQ, writing quality; TWW, total words written.

#### Aim 1: RWS model without fluency

Several effects were in the expected range in the reduced model without R-W fluency (Model 1; see Table 7). Word reading predicted handwriting (*β* = 0.39, SE = 0.06, *p* < 0.001), spelling (*β* = 0.76, SE = 0.04, *p* < 0.001), and writing quality (*β* = 0.22, SE = 0.09, *p* < 0.05). Vocabulary predicted spelling (*β* = 0.09, SE = 0.05, *p* < 0.05), editing (*β* = 0.20, SE = 0.05, *p* < 0.001), and writing quality (*β* = 0.11, SE = 0.05, *p* < 0.05). Reading strategies predicted editing (*β* = 0.44, SE = 0.05, *p* < 0.001), revision (*β* = 0.33, SE = 0.08, *p* < 0.001), and writing quality (*β* = 0.30, SE = 0.09, *p* < 0.001). As hypothesized, higher-order reading skills such as reading strategies were related to writing quality, but contrary to our expectations, inferencing (*β* = 0.14, SE = 0.10, *p* > 0.05) and knowledge (*β* = 0.06, SE = 0.05, *p* > 0.05) were not related to writing quality. Similarly, reading strategies were related to higher-order writing skills (editing and revision), but other higher-order reading skills (background knowledge and inferencing) were not related to planning, editing, or revising (see results for Model 1 in Table 7). Finally, reading comprehension predicted planning (*β* = 0.22, SE = 0.06, *p* < 0.001), editing (*β* = 0.18, SE = 0.05, *p* < 0.001), revision (*β* = 0.25, SE = 0.07, *p* < 0.001), and writing quality (*β* = 0.24, SE = 0.07, *p* < 0.001).

**Table 7:** Standardized solutions for the structural portion of the Reading-to-Writing Skills models.

	Model 1				Model 2			
	No fluency		ORF		SRF		ORF and SRF	
Parameter	Estimate	SE	Estimate	SE	Estimate	SE	Estimate	SE
*Word reading*								
β_WORD➔ HW_	0.39**	0.06	0.35**	0.05	0.35**	0.05	0.35**	0.05
β_WORD➔ SPELL_	0.76**	0.04	0.25	0.16	0.73**	0.03	0.25	0.17
β_WORD➔ WQ_	0.22*	0.09	0.37	0.26	0.23	0.17	0.36	0.26
β_WORD➔ TWW_	N/A	N/A	0.21	0.24	0.21	0.16	0.18	0.24
*Vocabulary*								
β_VOC➔ SPELL_	0.09*	0.05	0.07*	0.05	0.11*	0.04	0.07	0.05
β_VOC➔ PLAN_	0.02	0.06	0.01	0.06	0.02	0.06	0.01	0.06
β_VOC➔ EDIT_	0.20**	0.05	0.18**	0.04	0.09*	0.05	0.16*	0.06
β_VOC➔ REV_	0.06	0.06	0.05	0.05	0.004	0.06	0.03	0.07
β_VOC➔ WQ_	0.11*	0.05	0.12*	0.05	0.12	0.08	0.12	0.06
β_VOC➔ TWW_	N/A	N/A	0.002	0.05	−0.002	0.08	−0.02	0.07
*Βackground knowledge*								
β_ΒK➔ PLAN_	0.06	0.06	0.05	0.06	0.02	0.06	0.05	0.06
β_ΒK➔ WQ_	0.06	0.05	0.07	0.05	0.07	0.05	0.08	0.05
*Reading strategies*								
β_RS➔ PLAN_	0.09	0.07	−0.002	0.08	0.07	0.07	−0.002	0.08
β_RS➔ EDIT_	0.44**	0.05	0.22**	0.06	0.24**	0.07	0.20*	0.07
β_RS➔ REV_	0.33**	0.08	0.21*	0.08	0.23*	0.08	0.20*	0.08
β_RS➔ WQ_	0.30**	0.09	0.24*	0.08	0.27**	0.08	0.24**	0.08
*Inferencing*								
β_INF➔ REV_	−0.08	0.10	−0.11	0.10	−0.13	0.11	−0.13	0.11
β_INF➔ WQ_	0.14	0.10	0.10	0.10	0.15	0.11	0.10	0.11
*Reading comprehension*								
β_RC➔ PLAN_	0.22**	0.06	0.18*	0.07	0.23**	0.06	0.18*	0.07
β_RC➔ EDIT_	0.18**	0.05	0.12	0.05	0.05	0.06	0.11	0.06
β_RC➔ REV_	0.25**	0.07	0.24**	0.07	0.20*	0.08	0.23*	0.07
β_RC➔ WQ_	0.24**	0.07	0.32*	0.11	0.24*	0.08	0.31*	0.11
β_RC➔ TWW_	N/A	N/A	0.22*	0.09	0.22*	0.08	0.20*	0.10
*Oral reading fluency*								
β_ORF➔ SPELL_	N/A	N/A	0.50*	0.16	@0	@0	0.50*	0.17
β_ORF➔ PLAN_	N/A	N/A	0.14*	0.07	@0	@0	0.15*	0.08
β_ORF➔ EDIT_	N/A	N/A	0.31**	0.06	@0	@0	0.25*	0.10
β_ORF➔ REV_	N/A	N/A	0.18*	0.07	@0	@0	0.12	0.12
β_ORF➔ WQ_	N/A	N/A	−0.13	0.28	@0	@0	−0.12	0.28
β_ORF➔ TWW_	N/A	N/A	0.02	0.27	@0	@0	0.01	0.26
*Silent reading fluency*								
β_SRF➔ EDIT_	N/A	N/A	@0	@0	0.40**	0.09	0.09	0.14
β_SRF➔ REV_	N/A	≤N/A	@0	@0	0.23*	0.10	0.11	0.18
β_SRF➔ WQ_	N/A	N/A	@0	@0	−0.002	0.26	0.004	0.18
β_SRF➔ TWW_	N/A	N/A	@0	@0	0.02	0.23	0.08	0.16

**p ≤ 0.001; *p ≤ 0.05.

N/A, fluency variables were not included in the model. @0, path was constrained to 0 (i.e., it was not estimated).

#### Aim 2: RWS model with fluency

In the full Reading-to-Writing Skills model (Model 2, Figure 2) there were similarities in the pattern of associations with Model 1 and one notable difference. Word reading was still related to handwriting (*β* = 0.35, SE = 0.05, *p* < 0.05). Reading strategies still predicted editing (*β* = 0.20, SE = 0.07, *p* < 0.05), revision (*β* = 0.20, SE = 0.08, *p* < 0.05), and writing quality (*β* = 0.24, SE = 0.08, *p* < 0.05), and reading comprehension predicted planning (*β* = 0.18, SE = 0.07, *p* < 0.05), revision (*β* = 0.23, SE = 0.07, *p* < 0.05), and writing quality (*β* = 0.31, SE = 0.11, *p* < 0.05). However, vocabulary was only related to editing (*β* = 0.16, SE = 0.06, *p* < 0.05), and in this model ORF was related to spelling (*β* = 0.50, SE = 0.17, *p* < 0.05), planning (*β* = 0.15, SE = 0.08, *p* < 0.05), and editing (*β* = 0.25, SE = 0.10, *p* < 0.05). Writing productivity was predicted by reading comprehension (*β* = 0.20, SE = 0.10, *p* < 0.05), but not decoding, vocabulary, ORF, or SRF. As Table 7 shows, the nested model for SRF (Δχ^2^ (6) = 14.35, *p* < 0.05) was significantly worse fitting than the full model in terms of overall fit, whereas the model for ORF (Δχ^2^ (4) = 0.86, *p* > 0.05) was not significantly different from the full model, but these solutions pointed to a key difference: when the hypothesized direct effects of ORF on writing skills are not controlled for, SRF predicted the higher-order writing skills with heavier cognitive load, editing (*β* = 0.40, SE = 0.09, *p* < 0.001) and revising (*β* = 0.23, SE = 0.10, *p* < 0.05), whereas when the hypothesized direct effects of SRF on writing skills are not controlled for, ORF predicted the writing skills with lower (spelling; *β* = 0.50, SE = 0.16, *p* < 0.05) and higher cognitive load (planning, *β* = 0.14, SE = 0.07, *p* = 0.05; editing, *β* = 0.31, SE = 0.06, *p* < 0.05; and revising, *β* = 0.18, SE = 0.07, *p* < 0.05), and vocabulary predicted writing quality (*β* = 0.12, SE = 0.05, *p* < 0.05). However, when both ORF and SRF were evaluated simultaneously in the full model, only the effects of ORF remained statistically significant.

### Reading-to-Writing Mediation models

The reduced RWM model without R-W fluency (Figure 4) provided a good fit to the data (χ^2^ (*df*) = 202.80 (121), *p* < 0.001; RMSEA [90% CI] = 0.04 [0.03, 0.05]; CFI = 0.97; TLI = 0.95; SRMR = 0.04), as did the full RWM model (Figure 6; χ^2^ (*df*) = 368.22 (195), *p* < 0.001; RMSEA [90% CI] = 0.05 [0.04, 0.05]; CFI = 0.96; TLI = 0.94; SRMR = 0.04). Table 3 shows that the RWM models explained a larger proportion of variance compared to the direct effects models (e.g., the full RWM model explained 90% variance in writing quality). These models also explained a large amount of variance in reading skills (e.g., the full RWM model explained 62% variance in inferencing and 94% in ORF). The measurement model solutions were similar to the solution of the RWS models presented above (see Table 4).

#### Aim 3: RWM model without fluency

First, the results of the RWM model showed that several direct effects of R-to-R skills were in the expected range: inferencing (*β* = 0.48, SE = 0.14, *p* < 0.05) and background knowledge (*β* = 0.14, SE = 0.05, *p* < 0.05) predicted reading comprehension; vocabulary (*β* = 0.24, SE = 0.06, *p* < 0.001) and reading strategies (*β* = 0.53, SE = 0.08, *p* < 0.001) predicted inferencing; and word literacy predicted reading strategies (*β* = 0.64, SE = 0.06, *p* < 0.001). The effects of several W-to-W skills were also in the expected range: word literacy was related to handwriting (*β* = 0.43, SE = 0.05, *p* < 0.05) and editing (*β* = 0.59, SE = 0.10, *p* < 0.001); handwriting was related to planning (*β* = 0.18, SE = 0.06, *p* < 0.001), editing (*β* = 0.10, SE = 0.04, *p* < 0.05), and writing quality (*β* = 0.25, SE = 0.05, *p* < 0.001). Planning was related to revision (*β* = 0.11, SE = 0.05, *p* < 0.05) and writing quality (*β* = 0.11, SE = 0.04, *p* < 0.05), and editing was related to revision (*β* = 0.31, SE = 0.06, *p* < 0.001), which in turn was related to writing quality (*β* = 0.31, SE = 0.05, *p* < 0.001). Second, Table 8 shows that several cross-domain direct effects from the RWS model (Model 1) also remained significant (e.g., vocabulary ➔ editing, reading strategies ➔ writing quality, reading comprehension ➔ planning, reading comprehension ➔ revision, and reading comprehension ➔ writing quality), with some exceptions: reading strategies were no longer predictive of editing or revision. However, the total indirect effects of several variables were significant as the associations were driven by one or more mediators, as shown in Table 9. Three effects were partially mediated: the effects of reading strategies (*via* inferencing and reading comprehension), reading comprehension (*via* revision), and handwriting, on writing quality because zero was not included in the 95% confidence intervals for these effects (see Table 9). Five effects were completely mediated, indicating that the mediators explained all of the relationship between the variables: (1) vocabulary to reading comprehension (specifically, via inferencing), (2) reading strategies to reading comprehension (specifically, via inferencing), (3) inferencing to revision (specifically, via reading comprehension), (4) background knowledge to writing quality, and (5) editing to writing quality.

**Table 8:** Standardized solutions for the structural portion of the Reading-to-Writing Mediation (RWM) models.

Reduced RWM Model (Model 3)				Full RWM Model (Model 4)			
Path	Parameter	SE	Path #	Path	Parameter	SE	Path #
*Word literacy*				*Word literacy*			
β_WORD➔ HW_	0.43*	0.05	N1	β_WORD➔ HW_	0.40**	0.05	N1
β_WORD➔ EDIT_	0.59**	0.10	N2	β_WORD➔ EDIT_	2.09	2.33	N2
β_WORD➔ RS_	0.64**	0.06	D4	β_WORD➔ RS_	N/A	N/A	D4
β_WORD➔ RC_	0.22	0.10	D5	β_WORD➔ RC_	N/A	N/A	D5
				β_WORD➔ ORF_	0.94**	0.04	F1
				β_WORD➔ SRF_	0.66*	0.34	F2
				β_WORD➔ TWW_	0.001	10.97	C18
*Vocabulary*				*Vocabulary*			
β_VOC➔ EDIT_	0.16**	0.05	C2	β_VOC➔ EDIT_	0.28	0.37	C2
β_VOC➔ RS_	0.04	0.06	D6	β_VOC➔ RS_	−0.30	0.39	D6
β_VOC➔ INF_	0.24**	0.06	D7	β_VOC➔ INF_	−0.11	0.41	D7
β_VOC➔ RC_	0.06	0.06	D8	β_VOC➔ RC_	−0.01	0.70	D8
				β_VOC➔ ORF_	0.04	0.05	F3
				β_VOC➔ SRF_	0.27**	0.07	F4
*Background knowledge*				*Background knowledge*			
β_ΒK➔ WQ_	0.09	0.04	C7	β_ΒK➔ WQ_	−0.03	0.11	C7
β_BK➔ RS_	0.10	0.06	D9	β_BK➔ RS_	−0.12	0.37	D9
β_BK➔ INF_	0.13	0.07	D10	β_BK➔ INF_	−0.16	0.33	D10
β_BK➔ RC_	0.14*	0.05	D11	β_BK➔ RC_	0.08	0.45	D11
				β_ΒK➔ SRF_	0.21**	0.07	F5
*Reading strategies*				*Reading strategies*			
β_RS➔ INF_	0.53**	0.08	D12	β_RS➔ INF_	0.13	0.38	D12
β_RS➔ RC_	−0.04	0.16	D13	β_RS➔ RC_	−0.03	0.58	D13
β_RS➔ EDIT_	0.07	0.09	C8	β_RS➔ EDIT_	0.03	0.34	C8
β_RS➔ PLAN_	0.06	0.07	C9	β_RS➔ PLAN_	0.02	0.08	C9
β_RS➔ REV_	0.23	0.14	C10	β_RS➔ REV_	0.20	0.17	C10
β_RS➔ WQ_	0.32**	0.08	C11	β_RS➔ WQ_	0.19	0.14	C11
				β_RS➔ TWW_	0.07	0.36	C24
*Inferencing*				*Inferencing*			
β_INF➔ RC_	0.48*	0.14	D14	β_INF➔ RC_	0.13	0.33	D14
β_INF➔ REV_	−0.15	0.18	C12	β_INF➔ REV_	−0.12	0.21	C12
*Reading comprehension*				*Reading comprehension*			
β_RC➔ PLAN_	0.24**	0.06	C15	β_RC➔ PLAN_	0.21**	0.06	C15
β_RC➔ REV_	0.20*	0.18	C16	β_RC➔ REV_	0.18*	0.08	C16
β_RC➔ WQ_	0.22**	0.05	C17	β_RC➔ WQ_	0.12	0.07	C17
				β_RC➔ TWW_	0.11	0.10	C29
*Handwriting*				*Handwriting*			
β_HW➔ EDIT_	0.10*	0.04	N3	β_HW➔ EDIT_	0.11*	0.04	N3
β_HW➔ PLAN_	0.18**	0.06	N4	β_HW➔ PLAN_	0.17**	0.06	N4
β_HW➔ WQ_	0.25**	0.05	N5	β_HW➔ WQ_	0.23**	0.05	N5
				β_HW➔ TWW_	0.18	0.35	P1
*Plan*				*Plan*			
β_PLAN➔ EDIT_	0.05	0.04	N8	β_PLAN➔ EDIT_	0.06	0.05	N8
β_PLAN➔ REV_	0.11*	0.05	N9	β_PLAN➔ REV_	0.12*	0.05	N9
β_PLAN➔ WQ_	0.11*	0.04	N10	β_PLAN➔ WQ_	0.09*	0.05	N10
				β_PLAN➔ TWW_	0.12	0.20	P3
*Edit*				*Edit*			
β_EDIT➔ REV_	0.31**	0.06	N6	β_EDIT➔ REV_	0.32**	0.07	N6
β_EDIT➔ WQ_	0.05	0.06	N7	β_EDIT➔ WQ_	−0.03	0.08	N7
				β_EDIT➔ TWW_	−0.06	3.01	P2
*Revision*				*Revision*			
β_REV➔ WQ_	0.31**	0.05	N11	β_REV➔ WQ_	0.29**	0.05	N11
				β_REV➔ TWW_	0.19*	0.07	P4
				*Writing productivity*			
				β_TWW➔ WQ_	0.18**	0.05	P5
				*Oral reading fluency*			
				β_ORF➔ EDIT_	−1.47	2.34	C20
				β_ORF➔ PLAN_	0.09	0.07	C21
				β_ORF➔ REV_	0.03	0.19	C22
				β_ORF➔ WQ_	−0.23	0.22	C23
				β_ORF➔ TWW_	0.08	9.01	C24
				β_ORF➔ RS_	−0.39	1.14	F6
				β_ORF➔ RC_	0.13	1.62	F7
				β_ORF➔ INF_	−0.58	0.87	F8
				β_ORF➔ SRF_	0.06	0.35	F9
				*Silent reading fluency*			
				β_SRF➔ RS_	1.25	1.50	F10
				β_SRF➔ RC_	0.27	2.52	F11
				β_SRF➔ INF_	1.30	1.40	F12
				β_SRF➔ EDIT_	−0.17	0.96	C25
				β_SRF➔ REV_	0.01	0.28	C26
				β_SRF➔ TWW_	0.09	2.29	C27
				β_SRF➔ WQ_	0.52	0.34	C28

**p ≤ 0.001; *p ≤ 0.05.

Path # corresponds to paths in Figures 3–6. N/A, the path was excluded because the model did not converge with the inclusion of this path. Word literacy was significantly correlated with vocabulary (*r* = 0.31–0.33, *p* < 0.001) and background knowledge (*r* = 0.44–0.45, *p* < 0.001), and vocabulary and background knowledge were significantly correlated (*r* = 0.32–0.33, *p* < 0.001). In these models, several residual covariances were estimated to account for common method variance: (1) WJ-III Letter Word Identification and TOWRE Sight Word Efficiency (*r* = 0.26–0.35, *p* < 0.001); (2) WJ-III Spelling and percent words spelled correctly (*r* = 0.31–0.46, *p* < 0.001); and (3) WJ-III Letter Word Identification and WJ-III Spelling (*r* = 0.37, *p* < 0.001).

**Table 9:** Total indirect and specific indirect effects of the full Reading-to-Writing Mediation (RWM) models.

RWM Model 3				RWM Model 4			
Path	Parameter	SE	95% CI	Path	Parameter	SE	95% CI
Writing quality				Writing quality and productivity			
β_BK➔ WQ_	0.11**	0.03	[0.03, 0.18]	β_BK➔ WQ_	0.19*	0.10	[0.06, 0.85]
β_RS➔ WQ_^a^	0.13*	0.05	[0.01, 0.26]	β_RS➔ WQ_	0.07	0.12	[−1.67, 0.27]
β_RC➔ WQ_^b^	0.10*	0.04	[0.04, 0.20]	β_RC➔ WQ_	0.11*	0.04	[0.01, 0.21]
β_HW➔ WQ_	0.04*	0.01	[0.01, 0.09]	β_HW➔ WQ_	0.07**	0.02	[0.01, 0.11]
β_PLAN➔ WQ_	0.04*	0.02	[0.00, 0.09]	β_PLAN➔ WQ_	0.06*	0.02	[0.01, 0.12]
β_EDIT➔ WQ_	0.10**	0.03	[0.04, 0.18]	β_EDIT➔ WQ_	0.10	0.59	[−5.41, 1.53]
				β_ORF➔ WQ_	−0.10	0.45	[−4.33, 0.58]
				β_SRF➔ WQ_	0.45	0.56	[0.08, 10.22]
				β_WORD➔ TWW_	0.33	10.96	[−110.26, 26.57]
				β_VOC➔ TWW_	0.05	1.50	[−15.68, 5.99]
				β_ORF➔ TWW_	0.01	8.77	[−31.66, 55.39]
				β_SRF➔ TWW_	0.15	2.11	[−1.51, 11.08]
				β_HW➔ TWW_	0.03	0.35	[−3.86, 0.70]
				β_PLAN➔ TWW_	0.02	0.19	[−1.60, 0.78]
				β_EDIT➔ TWW_^g^	0.06	0.03	[0.01, 0.14]
Editing, planning, and revision				Editing, planning, and revision			
β_RS➔ PLAN_	0.05	0.03	[−0.01, 0.14]	β_RS➔ PLAN_	0.01	0.07	[−1.00, 0.09]
β_VOC➔ EDIT_	0.01	0.01	[−0.01, 0.05]	β_VOC➔ EDIT_	−0.10	0.36	[−1.69, 0.67]
β_RS➔ EDIT_	0.01	0.01	[−0.01, 0.04]	β_RS➔ EDIT_	0.01	0.01	[−0.02, 0.03]
β_HW➔ EDIT_	0.01	0.10	[−0.01, 0.04]	β_HW➔ EDIT_	0.01	0.01	[−0.01, 0.04]
β_RC➔ REV_	0.03*	0.01	[0.00, 0.09]	β_RC➔ REV_	0.03	0.02	[0.00, 0.08]
β_INF➔ REV_^c^	0.11	0.15	[0.03, 0.70]	β_INF➔ REV_	0.08	0.14	[−0.18, 0.46]
β_RS➔ REV_	−0.02	0.13	[−0.35, 0.18]	β_RS➔ REV_	0.01	0.16	[−1.21, 0.38]
β_PLAN➔ REV_	0.02	0.02	[−0.01, 0.06]	β_PLAN➔ REV_	0.02	0.02	[−0.02, 2.67]
				β_ORF➔ PLAN_	−0.02	0.09	[−0.41, 0.12]
				β_ORF➔ EDIT_	−0.02	0.95	[−9.36, 0.23]
				β_ORF➔ REV_	−0.47	0.67	[−6.41, 0.17]
				β_SRF➔ EDIT_	0.07	2.95	[−10.48, 3.41]
				β_SRF➔ REV_	0.19	0.74	[−0.49, 7.23]
Reading strategies, inferencing, and reading comprehension				Reading strategies, inferencing, reading fluency, and reading comprehension			
β_VOC➔ INF_	0.02	0.03	[−0.07, 0.11]	β_VOC➔ INF_	0.34	0.41	[0.06, 7.59]
β_BK➔ INF_	0.05	0.03	[−0.02, 0.15]	β_BK➔ INF_	0.30	0.32	[0.06, 4.90]
β_WORD➔ RC_	0.14*	0.07	[−0.07, 0.32]	β_WORD➔ RC_	0.47**	0.05	[0.35, 0.60]
β_VOC➔ RC_^e^	0.12*	0.04	[0.04, 0.28]	β_VOC➔ RC_	0.17	0.69	[−0.40, 12.37]
β_BK➔ RC_	0.08*	0.03	[−0.001, 0.23]	β_BK➔ RC_	0.11	0.45	[−0.38, 4.60]
β_RS➔ RC_^f^	0.25*	0.11	[0.10, 0.92]	β_RS➔ RC_	0.05	0.22	[−1.73, 0.29]
				β_VOC➔ SRF_	0.00	0.02	[−0.05, 0.09]
				β_ORF➔ INF_	0.04	0.84	[−1.95, 4.98]
				β_SRF➔ INF_	0.16	1.20	[−1.37, 7.07]
				β_ORF➔ RC_	−0.18	1.51	[−6.22, 3.51]
				β_SRF➔ RC_	0.53	2.24	[−0.92, 24.19]

*p < 0.05; **p < 0.001.

Statistically significant specific indirect effects: ^a^β_RS ➔ INF ➔ RC ➔ WQ_ = 0.06 [0.02, 0.22]; ^b^β_RC ➔ REV ➔ WQ_ = 0.06 [0.01, 0.16]; ^c^β_INF➔ RC ➔ REV_ = 0.10 [0.02, 0.66]; ^e^β_VOC➔ INF ➔ RC_ = 0.11 [0.04, 0.27]; ^f^β_RS ➔ INF ➔ RC_ = 0.25 [0.10, 0.92]; ^g^β_EDIT ➔ REV ➔ TWW_ = 0.06, 95% CI [0.01, 0.14].

#### Aim 4: RWM model with fluency

The full RWM model (Model 4) included all the variables from Model 3 and specified relations with R-W fluency (ORF, SRF, and total words written). In this model, additional R-to-R paths were in the expected range (word literacy predicted ORF [*β* = 0.94, SE = 0.04, *p* < 0.001] and SRF [*β* = 0.66, SE = 0.34, *p* < 0.05]; vocabulary [*β* = 0.27, SE = 0.07, *p* < 0.001] and background knowledge [*β* = 0.21, SE = 0.07, *p* < 0.001] predicted SRF, but vocabulary was not related to ORF [*β* = 0.04, SE = 0.05, *p* > 0.05]). Contrary to our expectations, ORF and SRF were not significant predictors of inferencing, strategies, or comprehension after controlling for all other variables in the model. Two additional effects of W-to-W skills were in the expected range: revision predicted productivity (*β* = 0.18, SE = 0.05, *p* < 0.05), and productivity predicted writing quality (*β* = 0.18, SE = 0.05, *p* < 0.001). Unlike the RWS Model 2, writing productivity was not significantly predicted by reading comprehension in the RWM model (see Table 8).

Table 8 shows that most cross-domain effects from the RWS model (Model 2) also remained significant, with some exceptions: background knowledge did not have a direct effect on writing quality, and ORF was no longer predictive of planning or editing. However, the total indirect effect of background knowledge to writing quality was significant as these variables were indirectly related through multiple variables in the R-W system (e.g., knowledge ➔ SRF ➔ writing quality, as well as knowledge ➔ SRF ➔ reading comprehension ➔ writing quality), but none of these specific indirect effects were statistically significant. Overall, Table 9 shows few indirect effects were statistically meaningful (i.e., did not include 0 in the confidence intervals). While editing was not directly related to writing productivity in the RWM model (see Table 8), this effect was completely mediated by revision (*β* = 0.06, 95% CI [0.01, 0.14]). Finally, nested models that evaluated the hypothesized relations of SRF with writing variables independently of the relation of ORF with writing variables (and vice versa) did not yield a different pattern of results.

### Alternative models

The Reading-to-Writing Domains model (alternative model 1) and the Writing-to-Reading Skills model (alternative model 2) provided a good fit to the data (e.g., CFI = 0.97, SRMR = 0.03; see Supplementary Appendixes A,B). The measurement models were similar to that of the RWS model, but the R-to-W Domains model also included a general factor for writing because all the writing dimensions and the CIWS shared method variance (i.e., required human ratings and were derived from the same written response). The R-to-W Domains model explained over half of the variance in writing dimensions (e.g., 83% for ideas; see Table 3). The model also explained 43% of the variance in CIWS. However, the R-to-W Domains model showed that multiple reading skills were not differentially related to specific writing dimensions, except for ORF, which predicted CIWS (*β* = 0.26, SE = 0.11, *p* < 0.05) but not the six traits (see Supplementary Appendix A). The nested models for ORF (Δχ^2^ (5) = 4.29, *p* > 0.05) and SRF (Δχ^2^ (4) = 5.55, *p* > 0.001) were not significantly different from the full model in terms of overall fit. The reduced model solutions again pointed to a key difference: when ORF did not make direct contributions to specific domains, word reading predicted word choice (*β* = 0.10, SE = 0.04, *p* < 0.05) and SRF also predicted conventions (*β* = 0.21, SE = 0.09, *p* < 0.05) and CIWS (*β* = 0.44, SE = 0.06, *p* < 0.001). When paths from SRF to writing domains were omitted, ORF predicted conventions (*β* = 0.23, SE = 0.12, *p* < 0.05) and CIWS (*β* = 0.37, SE = 0.05, *p* < 0.05).

The Writing-to-Reading Model specified regressions of reading skills on writing skills. The diagram and results of this model are presented in Supplementary Appendix B. Several effects were in the expected range: spelling predicted word reading (*β* = 0.61, SE = 0.07, *p* < 0.001), ORF (*β* = 0.59, SE = 0.07, *p* < 0.001), and SRF (*β* = 0.40, SE = 0.08, *p* < 0.05), and interestingly, spelling also predicted background knowledge (*β* = 0.21, SE = 0.07, *p* < 0.001); editing predicted vocabulary (*β* = 0.26, SE = 0.07, *p* < 0.001), inferencing (*β* = 0.23, SE = 0.08, *p* < 0.05), reading strategies (*β* = 0.19, SE = 0.07, *p* < 0.05), SRF (*β* = 0.11, SE = 0.07, *p* < 0.001), and reading comprehension (*β* = 0.17, SE = 0.06, *p* < 0.05); and writing quality predicted all reading skills, except for word reading. This model explained over half of the variance in reading strategies and ORF and SRF, and a smaller percent of variance in vocabulary and knowledge (see Table 3).

## Discussion

A better understanding of the connection of R-W is of vital importance for supporting struggling writers considering the continuing difficulties exhibited and the documented relationships among these skills. Students who experience reading difficulties are increasingly likely to also experience difficulties in writing but teachers report sideling evidence-based writing instruction in the classroom (Graham et al., 2014). Understanding the skill patterns between R-W in ways that support the identification of other skill areas of need is critical.

Increasingly there are theoretical models that highlight R-W connections given their overlap in use of skills (Costa et al., 2016). In this study, we joined DIME components with NVSW to evaluate how lower- and higher-order skills in one domain impact counterpart skills in the other domain, including both ORF and SRF in the models. We limited the scope of the RWM model to *malleable* skills (i.e., those amenable to training) to increase its practical utility. Thus, we excluded the executive function (including attention, working memory, cognitive control, motivation, and self-efficacy) components of the NSVW model because there is a lack of compelling evidence that executive function training improve academic outcomes or predict response-to-intervention (Fletcher et al., 2018). We found support for the relations among DIME skills: mainly, vocabulary and strategies predicted inferencing, and higher-order knowledge and inferencing predicted reading comprehension. However, with the addition of fluency to the model, vocabulary predicted SRF instead of inferencing, and knowledge also predicted SRF instead of comprehension in line with recent research on this model (e.g., Oslund et al., 2018). We also found support for associations among component skills derived from the NSVW model: mainly among word literacy, handwriting and editing, as well as among planning, editing, revision, and writing quality and productivity. In the next sections, we highlight the findings of the cross-domain associations and their alignment with prior research.

### Reading-to-Writing connections

#### Lower-order reading skills

The results revealed that decoding is related to transcription, specifically spelling and handwriting. This is consistent with previous research which has shown that word-level R-W are connected due to a shared set of skills and knowledge that influence both. Fitzgerald and Shanahan (2000) describe several universal text attributes that help explain the relationship between spelling and decoding, including letter knowledge, phonological and morphological awareness, and knowledge of the orthography of the language. Other studies have also shown that word reading is a correlate of spelling skills (e.g., Abbott et al., 2010), and that it can predict spelling in languages varying in orthographic transparency (Georgiou et al., 2020).

We hypothesized that decoding and vocabulary would not predict writing quality, after controlling for higher-order cognition and comprehension. However, we found that decoding played a role in writing quality, highlighting this fundamental connection for children with LD. Interestingly, the opposite direction did not hold in the W-to-R Model because writing quality predicted all reading skills except for decoding. Collectively, these findings suggest that decoding is an active self-regulatory process in writing, beyond higher-order self-regulation (e.g., editing). Further, when students can read words accurately and fluently, they are more likely to use those words correctly in their own writing, which can help them expand their vocabulary and spelling skills, leading to overall writing quality. The study also found that decoding predicted word choice, a specific component of composition, possibly due to shared knowledge of components involved in the R-W process and could reflect an artifact of print exposure. As students are exposed to print and words, and their meaning, they become stored in the mental lexicon, and thus, more accessible during the writing process. However, it’s possible that students may select words that they know how to spell, thus reinforcing a potential W-to-R pathway. Future research is needed to disentangle these different mechanisms and to provide a more comprehensive understanding of the relationship between decoding, encoding, and word choice in composition.

We found that vocabulary was related to writing quality and editing, but not planning or revision. This suggests that stronger vocabulary facilitates conveying the intended meaning and identifying and correcting errors of spelling and usage effectively, but may not necessarily help revise (for content, organization, tone, and syntax), or help organize and structure ideas before beginning to write. Surprisingly, reading comprehension, rather than vocabulary, was related to productivity, possibly because better vocabulary allows students to express ideas succinctly and precisely but may relate less to total words written than other productivity measures that account for accuracy. On the other hand, comprehension may facilitate understanding the ideas and concepts students are writing about and avoid errors and misunderstandings in their writing that could slow them down.

#### Higher-order reading skills

In our analysis, we looked at the role of higher-level reading skills, such as background knowledge, inferencing, and reading strategies. Our hypothesis was that knowledge is important for planning because writers need to verbalize their knowledge of a topic before they start writing to focus their writing (Tierney and Pearson, 1983). This was possibly not supported because inexperienced writers simply retrieve ideas prompted by the topic and translate them into text without purposeful engagement in planning, while experienced writers develop a set of goals and generate ideas from their knowledge to achieve these goals (Kucer, 1985). It is also possible that the knowledge assessment in our study affected the results (i.e., it measured knowledge from the reading passages but that was not specific to the writing task). Importantly, we found evidence of an indirect relationship between general knowledge and writing quality. This is not surprising given the importance of knowledge access, use, and generation during the writing process (e.g., Bereiter and Scardamalia, 1987; Allen et al., 2014; Kim, 2020), but our findings suggest that general knowledge influenced writing through its indirect effect on other literacy skills although not via any specific indirect path. Future research should evaluate general and topic-specific knowledge in the context of multiple literacy skills.

Contrary to our hypothesis, inferencing did not predict revision possibly because students with LD are not sophisticated in their revision process to use inferencing skills to detect errors in meaning. Limpo et al. (2013) found that revision skills increase from elementary to secondary grades, but in general, students detect and correct mechanical errors more than substantive meaning errors, and further, students are significantly better at correcting than detecting errors in stories with errors deliberately embedded in them. Nonetheless, we found support for complete mediation in which the effect of inferencing on revision was mediated by reading comprehension. It is possible that students who are better at making inferences while reading (i.e., making logical and reasonable assumptions based on information that is not explicitly stated in texts) are better able to understand the deeper meaning of a text and draw connections between different pieces of information. Understanding and interpreting written text effectively may, in turn, allow them to make revisions so their writing is more effective.

Lastly, reading strategies were related to editing, revising, and quality, but the findings did not support our hypothesis that strategies would relate to planning. The performance measure of reading strategies involved reading a passage to compose a summary; students also completed a survey of reading and learning strategies. More strategic students demonstrated better editing and revising skills and produced higher quality compositions, as expected, because editing and revising are strategic and involve self-monitoring, although research on this is limited. Self-regulation (a strategic process) also distinguishes novice from expert writers and employing more strategies during writing likely results in higher quality compositions. Although the measure focused on reading strategies, there may be overlap with writing strategies due to shared knowledge and skills between the two. Procedural knowledge, purposive information access, and goal-setting could all be influencing factors.

#### Oral and silent reading fluency

ORF predicted spelling possibly because letter-sound knowledge (Paige et al., 2019) and conceptual word knowledge (Zutell and Rasinski, 1989) underlie both component skills. Zutell and Rasinski (1986) found that the correlation of spelling was higher with oral reading accuracy than rate or prosody, further emphasizing the important role of phonological and orthographic knowledge above and beyond speed or expressiveness of reading aloud. Further, Paige and colleagues evaluated the opposite directionality (spelling ➔ ORF) in a sample of third graders at risk for LDs and found a small, non-hypothesized direct effect of spelling on oral reading fluency after controlling for word- and non-word reading but they did not estimate indirect effects via word and non-word reading. Although these finding contribute to our understanding of the complex interactions among reading fluency and spelling, further research is needed to better understand the underlying mechanisms and directionality of the observed relationships.

ORF also predicted the sentence-level writing component with less cognitive load (editing), whereas SRF predicted planning, which requires deeper processing (Alamargot et al., 2006). Interestingly, both ORF and SRF were related to reviewing, and neither were predictive of discourse-level writing outcomes (productivity and quality), suggesting that ORF/SRF are implicated in specific writing processes (De Smet et al., 2018; Conijn et al., 2022). However, it should be noted that there is a lack of research available to definitively interpret the results of ORF/SRF with writing processes and dimensions.

Finally, ORF/SRF were related to specific dimensions of writing (CIWS and conventions). Interestingly, when paths for both SRF/ORF were included, SRF no longer related to writing dimensions. However, when paths from ORF were excluded, both SRF and decoding predicted conventions, and word reading predicted word choice. These findings emphasize the differential relations of fluency skills with writing dimensions, further emphasizing (a) the importance of the construct of fluency and the connections between fluency in R-W, and (b) the value of ORF/SRF not only as indicators of overall reading but as broader language indicators that capture dimensions such as CIWS, which is thought to reflect both fluency and accuracy of writing. It is therefore not surprising that ORF/SRF were also related to conventions (Conijn et al., 2022), given its design to capture elements of capitalization and punctuation, as these elements are also captured in the CIWS metric. Although based on fluency, ORF was originally designed to serve as an overall indicator of reading performance (Deno, 2003), capturing fluency and related skills (e.g., vocabulary and reading comprehension). The intention of reading fluency to function in this capacity can be seen particularly in the outcomes for our alternative Reading-to-Writing Domains model. To further advance our understanding of the complex relationship between reading fluency and writing processes/domains, future research should aim to explore the co-development of these skills in students with LDs. This research could benefit from utilizing experimental measures and procedures borrowed from the discourse-processing literature, such as eye-tracking technology to investigate concurrent, silent reading and writing processes (Anson and Schwegler, 2012) in addition to standardized measures of ORF/SRF. Such methods can provide valuable insights into the underlying cognitive mechanisms involved in the development of reading fluency and writing skills and could ultimately inform more effective interventions for students with LDs.

#### Reading comprehension

Contrary to our expectations, we found that writing quality and productivity were not related to ORF and SRF, but rather to reading comprehension. This means that just knowing more words or reading with accuracy, speed, and expression does not automatically lead to a deeper level of written expression or self-regulatory writing activities. Similarly, we found that reading comprehension (and not ORF/SRF, inferencing or strategies) played a significant role in multiple indirect effects. First, we expected that higher-order reading would mediate the relationship between word/world knowledge and writing processes (e.g., vocabulary ➔ inference ➔ revision, or vocabulary ➔ strategies ➔ planning), as students with stronger foundational skills could apply those skills better in R-W activities like planning. However, higher-order skills were found to mediate other higher-order skills and writing processes or outcomes (e.g., strategies ➔ inference ➔ reading comprehension ➔ writing quality), although these effects were small. Revision mediated the relationship between reading comprehension and writing quality, suggesting that comprehension is related to writing quality because it facilitates making revisions. Inferencing and reading comprehension mediated the relationship between reading strategies and writing quality, indicating that reading strategies facilitate better inferencing and understanding of the text, which in turn improves writing.

Our findings also showed that adequate reading comprehension relates to planning, editing, and revision and writing quality and productivity. Stronger comprehension facilitates generating and organizing ideas, communicating thoughts effectively in writing, and analyzing one’s own writing to identify and correct errors. These findings demonstrate that the connection between reading comprehension and writing goes beyond shared content or domain knowledge. The comprehension-planning link points to the connection with procedural knowledge for accessing information purposely, setting goals, and analyzing. The comprehension-editing link points to the pragmatic knowledge of text attributes (words, syntax, usage). The comprehension-revision link points to meta-knowledge about written language (functions and purposes).

The results suggest knowledge and abilities in R-W skills may transfer across domains. Teaching a skill in one domain (e.g., reading comprehension) can directly impact another (e.g., written expression). Implicit R-W connections may also develop (e.g., improved reading comprehension through targeted inferencing instruction may lead to better writing and revision). These findings have important implications for targeted interventions, particularly when paired with screeners like ORF in the context of MTSS.

### Writing-to-Reading connections

The W-to-R model fit well and provided evidence for robust effects of spelling, editing, and writing quality on word/letter-, sentence-, and text-level reading. Although the reading skills of inferencing and knowledge did not predict writing quality, the opposite directions held in the W-to-R model, which is an important finding. Effective writing requires anticipating the reader’s potential inferences and identifying important themes and connections from knowledge to produce coherent text. Better writers likely produced clearer and logically consistent texts, although we did not score the essays for logical coherence. Similarly, editing predicted inferencing, but planning and revision were not predictive of reading skills, suggesting that editing (the ability to correct errors in spelling, grammar, punctuation, and syntax) facilitates meaning making processes (i.e., inferencing) because both skills require detecting inconsistencies in meaning using context clues and background knowledge whereas planning does not necessarily rely on context cues. Revision involves making changes to the content or structure of text, which may not necessarily rely on the use of context clues or background knowledge in the same way as editing. Our findings suggest that writing activities (e.g., writing-to-learn) may build word/world knowledge and meaning-making processes (e.g., inferencing and comprehension monitoring) by providing opportunities to write about new topics, using new vocabulary, and monitoring inconsistencies and meaning in their texts. Additional research is needed to fully understand the W-to-R directionality in multiple instructional contexts (e.g., integrated R-W instruction using writing-to-learn or reading-to-write approaches) in ways that direct both theory and practice for specific populations like those with LDs.

### Limitations and future directions

The findings of this study are limited to students with/at-risk for LDs and should be replicated and systematically contrasted with both typically developing and other special populations. Further, the nature of the measures impacted our results. For example, planning was not affected by knowledge, possibly because the knowledge measure was not aligned with the writing prompt. The planning measure may have been insufficient to capture the student’s necessary background knowledge, as students were only given 5 min to plan without explicit instructions or tools. Also, writing requirements in most strategy assessments may align more with editing and revision than with planning. Alternative performance measures, such as comprehension monitoring, and the inclusion of writing-specific self-reported strategy use could potentially yield different results. In the RWM Model specifically, editing and revision are framed as activities that take place during writing, but their measures were based on a TOWL subtest that assessed the use of contextual conventions in student essays. Future research should incorporate experimental indicators for capturing inter- and intra-individual editing and revision patterns alongside offline measures of grammar, syntax, and organization, for example. It is possible that sentence-level measurement of SRF is insufficient and alternative indicators are necessary to capture the SRF needed for advanced text reading and composition. Similarly, we measured handwriting quality, not fluency (the ability to write quickly and legibly). Future research using measures of handwriting fluency could help further our understanding of how handwriting accuracy and rate impact associations among R-W, particularly in conjunction with ORF, SRF, and writing productivity. Lastly, regarding measurement used in the study, the individual traits of the 6 + 1 rubric have poor reliability and multicollinearity issues, as shown by high correlations among the traits in the present study (*r* = 0.53–0.81). Future research should assess writing dimensions using measures/indicators with high content and face validity. Future studies should also aim to include multiple prompts, genres, and types of measures and evaluate the common method variance in component skills models that make use of a single writing prompt to derive multiple indicators.

Another limitation is that insufficient data were available on the specific components, frequency, and duration of the writing instruction each student received. Bi-directional relations among malleable component-skills, such as those in the R-to-W models of the present study, should be systematically evaluated by introducing variations in (a) instruction and (b) executive function and motivation requirements of the task. For example, the DIME model’s direct/indirect relations changed as a function of the focus of intervention (e.g., foundational skills vs. text processing; Ahmed et al., 2022b). We expect that means in R-W skills, and pattern of direct/indirect effects in R-to-W and W-to-R models will be disrupted as a function of the instruction (e.g., writing-for-reading, text-structure) and task requirements. Future research is needed to evaluate the proposed model and alternative specifications (including W-to-R) in multiple instructional and assessment contexts.

## Conclusion

Despite existing evidence of the general connection between reading and writing for children with learning difficulties, an evaluation of the specific relationships between different literacy sub-skills is crucial for identifying potential areas for improvement and understanding instructional challenges. This study investigated higher-order reading skills of the Direct and Inferential Mediation (DIME) model as mediators between basic literacy and knowledge, and the writing processes and products of the Not-so-Simple View of Writing (NSVW) model. This study offers a comprehensive evaluation of connections among higher-order skills in both reading and writing domains in component-skills models, considering a range of model constellations that systematically explore the role of oral and silent reading fluency and comprehension skills. The three reading-to-writing models evaluated in the current study (Reading-to-Writing Skills, Reading-to-Writing Dimensions, and Reading-to-Writing Mediation) and the Writing-to-Reading alternative models are comprehensive in scope (in terms of the number of higher-order skills included) and depth (in terms of the granularity of constructs). The findings provide ongoing support for the importance of the constructs of higher-order reading and writing, and fluency, and add to the empirical literature on the direct and indirect effects among components skills at the sub-word/word, sentence, and text-levels. Specifically, the study’s findings highlight the intricate interplay between reading and writing skills, emphasizing the explicit roles of decoding, comprehension, fluency, and strategies in shaping writing quality and productivity, and the implicit roles of background knowledge and inferencing in shaping writing processes and quality. We found that, as opposed to vocabulary, decoding predicted word choice, and reading comprehension had an impact on writing productivity. Background knowledge exerted an indirect influence on writing through its effects on other literacy skills, and we identified a mediation effect of inferencing impacting revision through reading comprehension. Additionally, distinctions between oral and silent reading fluency emerged, with the former predicting spelling and editing and the latter predicting planning. Both types of fluency were linked to reviewing and specific dimensions of writing. Finally, writing quality was found to predict inferencing and knowledge, while editing predicted inferencing. These findings highlight the importance of fostering strong reading abilities and writing skills to enhance students’ overall literacy performance, emphasizing the need for a comprehensive approach to literacy education that nurtures both reading and writing competencies. Studying the relationship between reading fluency and writing skills across different levels of language is critical, given the reliance on ORF for screening and progress monitoring in practice. The study’s findings suggest that ORF may serve as an indicator of both reading difficulty and writing performance across different levels of writing. However, the results of the present study should serve as the basis for further studies on the relationship between reading fluency and component skills of writing.

## Data availability statement

The data are available upon request from the Texas Institute for Measurement, Evaluation, and Statistics (TIMES). Requests to access these datasets should be directed to yusra.ahmed@times.uh.edu.

## Ethics statement

The studies involving human participants were reviewed and approved by the Institutional Review Boards of the University of Houston and the University of Texas at Austin. Written informed consent to participate in this study was provided by the participants’ legal guardian/next of kin.

## Author contributions

YA conceived of the presented idea and performed the computations. YA, SCK, and MKM refined the ideas, discussed the results, and contributed to the final manuscript.

## Funding

This work was supported by the Eunice Kennedy Shriver National Institute of Child Health and Human Development (NICHD) under Grants R01HD096262 and P50HD052117.

## Conflict of interest

The authors declare that the research was conducted in the absence of any commercial or financial relationships that could be construed as a potential conflict of interest.

## Publisher’s note

All claims expressed in this article are solely those of the authors and do not necessarily represent those of their affiliated organizations, or those of the publisher, the editors and the reviewers. Any product that may be evaluated in this article, or claim that may be made by its manufacturer, is not guaranteed or endorsed by the publisher.

## Author disclaimer

The claims expressed in this article are solely those of the authors and do not necessarily represent those of the National Institute of Child Health and Human Development.

## Footnotes

^1^Additional writing dimensions were evaluated in the Reading-to-Writing Dimensions model (see Supplementary Appendix A).

^2^*Full models* refer to structural models that include all the R-W constructs we evaluated (14 or 15 observed and latent variables). This is different from fully saturated models, which outline every possible path among the constructs. Thus, the term *full* here refers to the number of factors rather than the number of paths.
