# Supplementary material for: Reading-to-Writing Mediation model of higher-order literacy
Source: Front Psychol. 2023 Jun 30;14:1033970. doi: 10.3389/fpsyg.2023.1033970 (PMC10349349; doi:10.3389/fpsyg.2023.1033970)
Supplement: Supplementary file 1 [file Data_Sheet_1.pdf]

APPENDIX A

Reading-to-Writing Dimensions Model

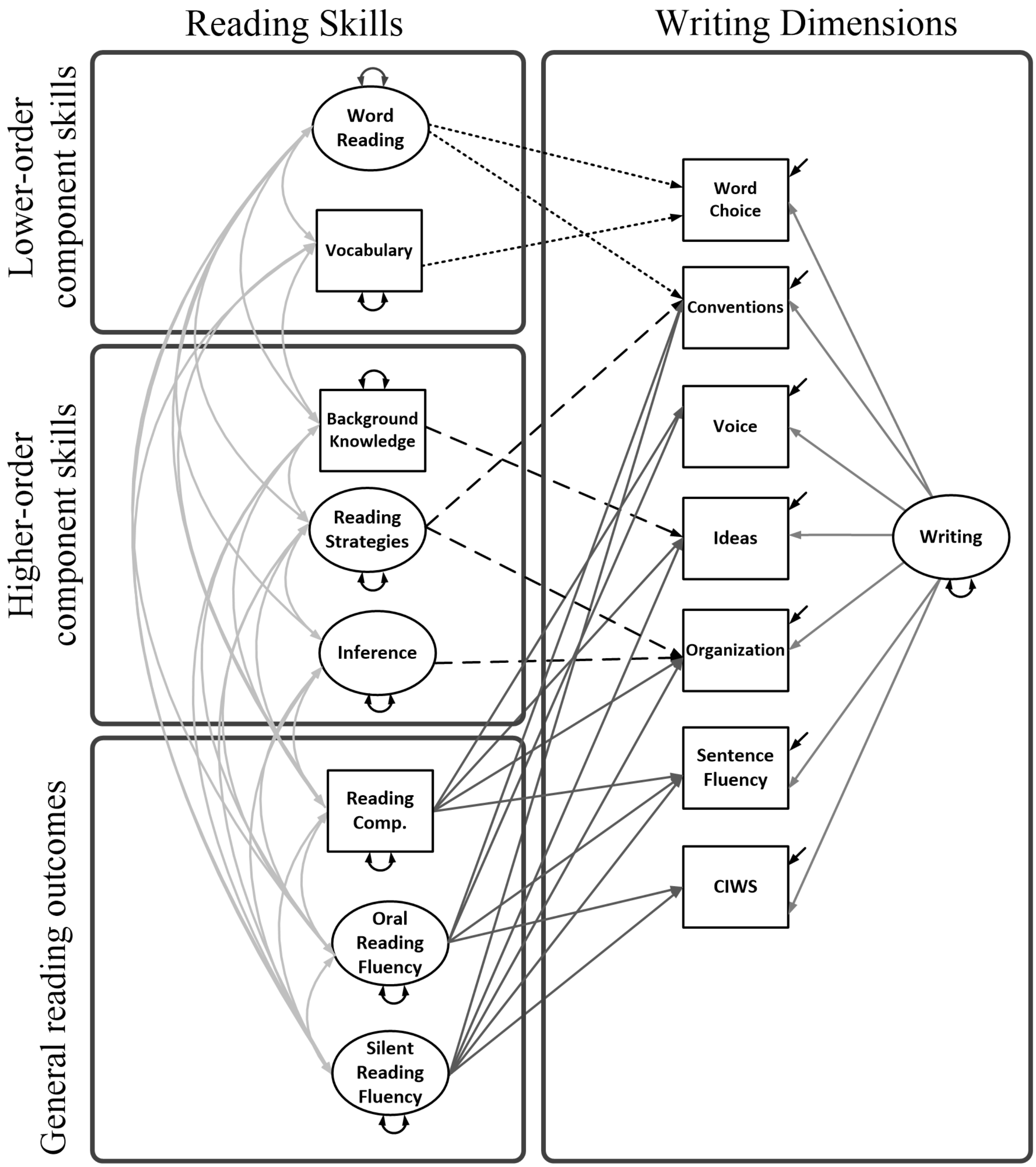

Note. Small-dashed lines are 3 paths from lower-order reading skills to writing dimensions; long-dashed lines are 4 paths from higher-order reading skills to writing dimensions; solid lines are 11 paths from reading outcomes to writing dimensions or 7 paths from a general writing dimension to specific writing dimensions. Double headed arrows are correlations. Correlations among Writing and Reading Skills were estimated but are not depicted in the

figure. Model fit indices: χ^2 (*df*) = 281.69 (155), $p < 0.001$; AIC = 19524.30; BIC = 20004.76; Sample-size adjusted BIC = 19623.99; RMSEA [90% CI] = 0.05 [.04, .05]; CFI = 0.97; TLI = 0.96; SRMR = 0.03.

Table S1. Standardized solution for the measurement model of the Reading-to-Writing Dimensions model.

Variable	Parameter	SE
Word Reading		
TOWRE SWE	0.84**	0.03
WJ LWID	0.71**	0.03
Reading Strategies		
CLS: Strategies	0.13*	0.06
Summary 1	0.71**	0.04
Summary 2	0.82**	0.03
Summary 3	0.67**	0.04
Inference		
Bridge-It Near	0.72**	0.03
Bridge-It Far	0.73**	0.03
Sentence Reading Fluency		
TOSREC 1	0.72**	0.03
TOSREC 2	0.73**	0.03
Oral Reading Fluency		
AIMSweb 1	0.92**	0.01
AIMSweb 2	0.91**	0.01
Writing		
Word Choice	0.77**	0.04
Conventions	0.55**	0.06
Organization	0.92**	0.05
Sentence Fluency	0.69**	0.05
Voice	0.90**	0.04
Ideas	0.95**	0.05
CIWS	0.33**	0.06

** $p < 0.001$, * $p < 0.05$.

Table S2. Standardized Solutions for the Reading-to-Writing Dimensions Models

	ORF & SRF		SRF		ORF	
**Parameter**	**Estimate**	**SE**	**Estimate**	**SE**	**Estimate**	**SE**
Word Reading						
$\beta_{WORD \rightarrow WC}$	0.09	0.06	0.10*	0.04	0.06	0.04
$\beta_{WORD \rightarrow CONV}$	0.01	0.12	0.09	0.07	-0.02	0.11
Vocabulary						
$\beta_{VOC \rightarrow WC}$	0.04	0.04	0.04	0.04	0.03	0.04
Background Knowledge						
$\beta_{BK \rightarrow IDEAS}$	-0.01	0.03	-0.01	0.03	-0.02	0.03
Reading Strategies						
$\beta_{RS \rightarrow CONV}$	0.01	0.06	-0.03	0.06	0.01	0.06
$\beta_{RS \rightarrow ORG}$	0.002	0.06	-0.06	0.05	-0.02	0.05
Inferencing						
$\beta_{INF \rightarrow ORG}$	-0.10	0.07	-0.09	0.07	-0.06	0.06
Reading Comprehension						
$\beta_{RC \rightarrow VOICE}$	-0.05	0.06	-0.05	0.05	-0.05	0.06
$\beta_{RC \rightarrow IDEAS}$	-0.09	0.06	-0.08	0.06	-0.09	0.05
$\beta_{RC \rightarrow ORG}$	-0.04	0.06	-0.05	0.06	-0.02	0.06
$\beta_{RC \rightarrow SF}$	-0.03	0.06	-0.04	0.06	-0.02	0.06
Oral Reading Fluency						
$\beta_{ORF \rightarrow CONV}$	0.17	0.16	@0	@0	0.23*	0.12
$\beta_{ORF \rightarrow VOICE}$	-0.003	0.07	@0	@0	-0.04	0.04
$\beta_{ORF \rightarrow SF}$	0.01	0.09	@0	@0	0.03	0.05
$\beta_{ORF \rightarrow CIWS}$	0.26*	0.11	@0	@0	0.37**	0.05
Silent Reading Fluency						
$\beta_{SRF \rightarrow CONV}$	0.08	0.12	0.21*	0.09	@0	@0
$\beta_{SRF \rightarrow IDEAS}$	0.03	0.09	0.03	0.06	@0	@0
$\beta_{SRF \rightarrow ORG}$	0.13	0.10	0.14*	0.07	@0	@0
$\beta_{SRF \rightarrow SF}$	0.07	0.12	0.11	0.07	@0	@0
$\beta_{SRF \rightarrow CIWS}$	0.17	0.13	0.44**	0.06	@0	@0

** $p \leq 0.001$; *$p \leq 0.05$.

Note. @0= path was constrained to 0 (i.e., it was not estimated).

APPENDIX B

Writing-to-Reading Model

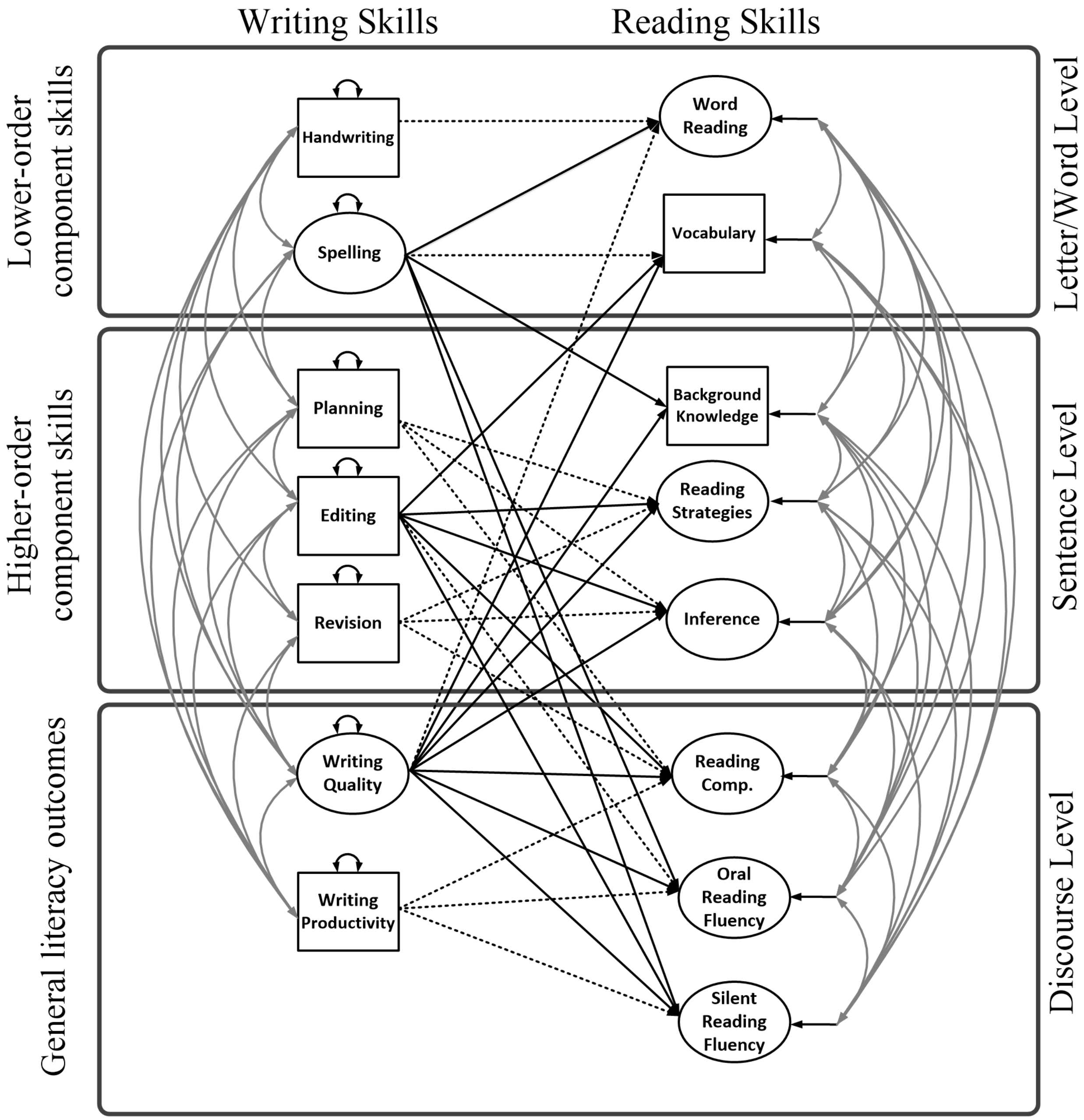

Note. Dashed lines were not statistically significant. All other paths from writing skills to reading skills were statistically significant. Model fit indices: χ^2 (*df*) = 296.74 (182), $p < 0.001$; AIC = 23077.41; BIC = 23645.96; Sample-size adjusted BIC = 23195.37; RMSEA [90% CI] = 0.04 [.03, .05]; CFI = 0.97; TLI = 0.96; SRMR = 0.03.

Table S3. Standardized Solution for the Writing-to-Reading Model

Measurement Model

Variable	Parameter	SE
Spelling		
WJ Spelling	0.88**	0.03
%WSC	0.75**	0.02
Word Reading		
TOWRE SWE	0.88**	0.03
WJ LWID	0.75**	0.02
Oral Reading Fluency		
AIMSweb 1	0.92**	0.01
AIMSweb 2	0.91**	0.01
Silent Reading Fluency		
TOSREC 1	0.73**	0.03
TOSREC 2	0.73**	0.03
Inference		
Bridge-It Near	0.78**	0.05
Bridge-It Far	0.52**	0.05
Reading Strategies		
CLS: Strategies	0.13*	0.06
Summary 1	0.70**	0.04
Summary 2	0.82**	0.03
Summary 3	0.67**	0.04

Structural Model

Path	Parameter	SE
Word Reading		
$\beta_{HW \rightarrow WR}$	0.02	0.05
$\beta_{SPELL \rightarrow WR}$	0.61**	0.07
$\beta_{WQ \rightarrow WR}$	0.14	0.09
Vocabulary		
$\beta_{SPELL \rightarrow VOC}$	0.02	0.08
$\beta_{EDIT \rightarrow VOC}$	0.26**	0.07
$\beta_{WQ \rightarrow VOC}$	0.18**	0.08
Background Knowledge		
$\beta_{WQ \rightarrow BK}$	0.30**	0.07
$\beta_{SPELL \rightarrow BK}$	0.21**	0.07
Reading Strategies		
$\beta_{PLAN \rightarrow RS}$	-0.10	0.06
$\beta_{EDIT \rightarrow RS}$	0.19*	0.07
$\beta_{REV \rightarrow RS}$	-0.07	0.08
$\beta_{WQ \rightarrow RS}$	0.66**	0.10
Inference		
$\beta_{PLAN \rightarrow INF}$	-0.02	0.06
$\beta_{EDIT \rightarrow INF}$	0.23*	0.08
$\beta_{REV \rightarrow INF}$	-0.16	0.08
$\beta_{WQ \rightarrow INF}$	0.61**	0.11
Reading Comprehension		
$\beta_{PLAN \rightarrow RC}$	0.03	0.04
$\beta_{EDIT \rightarrow RC}$	0.17*	0.06
$\beta_{REV \rightarrow RC}$	-0.02	0.06
$\beta_{WQ \rightarrow RC}$	0.52**	0.09
$\beta_{TWW \rightarrow RC}$	0.03	0.05
Oral Reading Fluency		
$\beta_{SPELL \rightarrow ORF}$	0.59**	0.07
$\beta_{EDIT \rightarrow ORF}$	-0.03	0.05
$\beta_{WQ \rightarrow ORF}$	0.20**	0.08
$\beta_{TWW \rightarrow ORF}$	0.08	0.04
Sentence Reading Fluency		
$\beta_{SPELL \rightarrow SRF}$	0.40**	0.08
$\beta_{EDIT \rightarrow SRF}$	0.11**	0.07
$\beta_{WQ \rightarrow SRF}$	0.32**	0.10
$\beta_{TWW \rightarrow SRF}$	0.04	0.06

** $p \leq 0.001$; $p \leq 0.05$.

APPENDIX C

Standardized Results for a Two-Factor Confirmatory Analytic Model (CFA) for the TOWL-4 Editing and Revision Subscales

TOWL-4 Contextual Conventions Items		Editing Loading (SE)	Revision Loading (SE)
**Editing**			
1	Sentences begin with a capital letter	0.56 (0.06)**	
3	Uses quotation marks	0.62 (0.07)**	
4	Uses comma to set off a direct quotation	0.52 (0.11)**	
5	Correctly uses an apostrophe at least once	0.60 (0.07)**	
6	Uses a question mark (?)	0.28 (0.12)*	
7	Uses an exclamation point (!)	0.44 (0.07)**	
8	Capitalizes proper nouns including those in story's title	0.39 (0.07)**	
9	Number of nonduplicated misspelled words (scored as 0 if there are 6 or more misspelled words)	0.40 (0.07)**	
10	Uses asterisk, ellipse, hyphen, parentheses, brackets	0.33 (0.10)**	
16	Noun-verb disagreements. E.g., They was running	0.34 (0.06)**	
19	Number of correctly spelled words having seven or more letters (count a word only once)	0.67 (0.05)**	
20	Number of words with three syllables or more that are spelled correctly	0.57 (0.08)**	
21	Uses a and an appropriately	0.32 (0.07)**	
**Revision**			
2	Paragraphs (clearly indicates paragraphs with indentations or spaces between)		0.46 (0.08)**
11	Fragmentary sentence - Usually a sentence without both a subject and a verb		0.49 (0.07)**
12	Run-on/Rambling sentence		0.55 (0.07)**
13	Compound sentences - Two complete sentences connected by a conjunction, colon, or semicolon; both sentences must have a subject and a verb		0.67 (0.05)**
14	Uses coordinating conjunctions other than and (but, or, nor, for, yet, so) when forming compound sentences; count each conjunction only once.		0.55 (0.07)**
15	Introductory phrases and clauses (Two or more words introducing a sentence; need not be set off by a comma.)		0.54 (0.06)**
17	Sentences in paragraph(s). E.g., 2 or more paragraphs and 2 or more sentences in at least 2 paragraphs		0.60 (0.06)**
18	Sentence composition. E.g., a variety of well-constructed compound and complex sentences		0.90 (0.04)**

Note. Model fit indices for the 2-factor model: χ^2 (*df*) = 263.06 (182), $p < 0.001$; RMSEA [90% CI] = 0.04 [.03, .04]; CFI = 0.98; TLI = 0.97; SRMR = 0.09. Residual variances were correlated for the following items because they were thematically related: 2 and 17; 19 and 20; 19 and 9; 9 and 20; 3 and 4; 13 and 14. The Modification Indices did not indicate the need to specify cross-loadings of Editing items on Revision, and Revision items on Editing. The 2-factor model was significantly different and provided a better fit to the data than a unidimensional model: Satorra-Bentler $\Delta\chi^2$ (Δdf) = 177.00 (7), $p < 0.01$.
